# Prenatal exposure to metal(loid)s mixture and childhood lung function: Exploring sex-specific associations

**DOI:** 10.1097/EE9.0000000000000447

**Published:** 2025-12-03

**Authors:** Mayra J. Garza, Cecilia S. Alcala, Marcela Tamayo-Ortiz, Adriana Mercado-Garcia, Nadya Y. Rivera Rivera, Chris Gennings, Martha María Téllez-Rojo, Robert O. Wright, Rosalind J. Wright, Héctor Lamadrid-Figueroa, María José Rosa

**Affiliations:** a Department of Perinatal Health, Center for Population Health Research, National Institute of Public Health (INSP), Cuernava, Morelos, Mexico; b Department of Environmental Medicine, Icahn School of Medicine at Mount Sinai, New York, New York; c Department of Environmental Health Sciences, Columbia University Mailman School of Public Health, New York, New York; d Center for Nutrition and Health Research, National Institute of Public Health, Cuernavaca, Morelos, Mexico; e Department of Public Health, Icahn School of Medicine at Mount Sinai, New York, New York; f Institute for Exposomic Research, Icahn School of Medicine at Mount Sinai, New York, New York

**Keywords:** Metals, Early-life exposure, Mixtures, Children’s health, Epidemiology

## Abstract

**Background:**

The link between prenatal exposure to metal(loid)s and childhood lung function, including sex-specific effects, has not been completely elucidated. We aimed to examine sex-specific individual and joint effects of prenatal metal(loid)s exposure on children’s lung function.

**Methods:**

Analyses included 438 mother-child dyads from the Programming Research in Obesity, Growth, Environment, and Social Stressors birth cohort in Mexico City. Metal(loid)s levels (As, Cd, Co, Cu, Mn, Ni, Pb, Se, and Zn) were measured in maternal blood collected during the second and third trimesters. Lung function in children was assessed once at ages 8–14 years. Multiple linear regression was performed to evaluate individual associations. Mixture effects were assessed through repeated holdout weighted quantile sum regression and hierarchical Bayesian kernel machine regression. Effect modification by sex was examined.

**Results:**

Second-trimester Ni was inversely associated with FEV_1_/FVC ratio in the overall sample and in females, while Pb was linked to lower FEV_1_ z-score in females. Third-trimester Co and Pb were also negatively associated with FVC z-score in females, with Pb also associated with lower FEV_1_ z-score in females. Higher metal mixture concentrations were associated with lower FEV_1_/FVC ratio (β = −0.33, 95% confidence interval: −0.48, −0.16) and FEF_25%–75%_ z-score (β = −0.22, 95% confidence interval: −0.39, −0.07) in females, with similar sex-specific patterns of association in weighted quantile sum and Bayesian kernel machine regression models.

**Conclusions:**

Second-trimester metal mixture exposure is associated with lower childhood lung function; females are potentially more susceptible to these exposures, which underscores the need to understand critical windows of exposure and sex-specific differences.

**What does this study add?**

By addressing how simultaneous prenatal exposure to metal(oid)s influences respiratory outcomes, this study provides novel insights into early-life environmental determinants of child health. We examined sex-specific individual and joint effects of prenatal exposure to metal(loid)s on lung function—specifically FEV_1_, FVC, FEV_1_/FVC ratio, and FEF_25%–75%_—using data from the well-characterized PROGRESS birth cohort in Mexico City. Higher exposure to metal mixtures during the second trimester of pregnancy was associated with lower lung function in childhood, particularly among females, who appeared more susceptible to these exposures.

## Introduction

Prenatal exposure to environmental factors has been implicated in influencing lung health and disease trajectory throughout the life course.^1,2^ Lung development begins *in utero* and continues postnatally, with the most rapid structural changes occurring during the fetal period.^2,3^ The second and third trimesters represent critical windows in which conducting airways, preacinar vessels, primitive alveoli, terminal bronchioles, type I and II cells, and surfactant production are developing.^4^ Insults during these periods, such as exposure to environmental toxicants, can disrupt these processes and result in impaired lung function.^4–6^ Reduced lung function serves as an early predictor not only of respiratory diseases but also for the development of cardiovascular disease, metabolic disorders, and premature mortality.^7–11^ Despite this understanding, significant gaps persist in our knowledge regarding the impact of perinatal factors on lung health across the lifespan.^1^

Exposure to metal(loid)s poses a public health threat as they accumulate in fetal tissues after crossing the placental barrier,^12–14^ in addition to their widespread presence in food, water, air, and soil across the world.^15^ Metal(loid)s may impair lung function by increasing oxidative stress, disrupting barrier mechanisms, causing inflammation, and tissue damage in the lungs^16^; which can result in decreased lung function postnatally and increased vulnerability to lung diseases in adulthood, such as asthma and chronic obstructive lung diseases.^17^ Furthermore, both toxic metal(loid)s (arsenic [As], cadmium [Cd], nickel [Ni], and lead [Pb]) and essential micronutrients (cobalt [Co], manganese [Mn], zinc [Zn], copper [Cu], and selenium [Se]) are commonly encountered from natural and anthropogenic sources.^18–23^ These elements may interact biologically,^24^ and several have well-documented adverse effects on the respiratory system.^23,25,26^

Despite the ubiquity and toxicity of metal(loid)s exposure, there is limited research on the impact of *in utero* exposure to metal(loid)s on lung function in children.^13,27–30^ Pregnant women are simultaneously exposed to numerous environmental contaminants, including toxic metals and essential nutrient elements with potential synergistic, additive, or antagonistic effects.^13,24^ Increasing evidence indicates that the toxicity of a chemical may be influenced by the presence of other chemicals, even at concentrations below their respective no observable adverse effect levels.^24^ Signes-Pastor et al^13^ reported no significant overall associations between prenatal urinary metal mixtures and children’s lung function at age ~7 using Bayesian kernel machine regression (BKMR). Our group recently reported associations between tooth metals concentrations during late gestation (at 12–15 weeks before birth) and reduced FVC z-scores in children aged 8–14 years.^18^

Furthermore, sex differences in susceptibility to metal exposure^31–33^ and fetal lung development^34^ have also been reported. Our group observed that the association between second trimester Pb levels and childhood FEV_1_/FVC ratio was modified by both child’s sex and maternal cortisol levels during pregnancy, suggesting differential sex-specific effects.^29^ However, to our knowledge, only two other studies have evaluated the relationship between exposure to metal mixtures during pregnancy and childhood lung function, exploring sex-specific effects.^18,30^

Consequently, by assessing metals' joint effects rather than individual associations, as well as sex-specific effects, this study will provide novel insights into early-life environmental determinants of child health. Thus, we utilized data from an ongoing birth cohort to investigate sex-specific individual and joint effects of exposure to metal(loid)s during pregnancy on children’s lung function parameters. We hypothesize that higher concentrations of prenatal metal(loid)s (hereinafter referred to as “metals”) in maternal blood are linked to lower lung function in childhood and will be modified by child sex.

## Methods

### Study population

Pregnant women receiving prenatal care at the Mexican Social Security Institute (Instituto Mexicano del Seguro Social) were enrolled in the Programming Research in Obesity, Growth, Environment and Social Stressors (PROGRESS) study from July 2007 to February 2011.^35^ To be eligible to participate, women needed to be <20 weeks of gestation, plan to remain in Mexico City for the next 3 years, ≥18 years old, have access to a phone, no daily consumption of alcohol, have no history of heart or kidney disease, and not be taking steroids or antiepileptic medications. Children’s lung function parameters were evaluated between the ages of 8 and 14, with 520 children completing testing, and after review by a pediatric pulmonologist, 449 (86%) met the standards for acceptability and reproducibility.^36^ A comprehensive flow diagram of participants included in the analysis is shown in Figure S1; https://links.lww.com/EE/A393. The study was approved by the Institutional Review Boards of the Icahn School of Medicine at Mount Sinai and the Mexican National Institute of Public Health (#642-2022; September 28, 2022), as well as by the Ethics Committee of the Mexican National Institute of Public Health (#1809; September 1, 2022). Written informed consent was obtained from participating women, while assent was provided by their children.

### Prenatal metals assessment

Maternal blood samples were collected at the second and third trimester visit. All blood samples were collected in trace metal-free tubes and stored at a temperature of 2–6 °C until analysis. The analyzed metals included As, Cd, Co, Cu, Mn, Ni, Pb, Se, and Zn following the procedure previously described for Pb.^37^ In summary, blood samples were digested using concentrated nitric acid (HNO₃) and 30% hydrogen peroxide (H₂O₂), then analyzed with an Agilent 8800 ICP Triple Quad instrument (Agilent Technologies, Inc., Santa Clara, CA) in tandem mass spectrometry mode. Previous publications^38–40^ outline additional quality control procedures. Metal concentrations were log_2_-transformed for analyses.

### Pulmonary function testing

Before testing, the child’s height and weight were measured using a stadiometer and an electronic scale, respectively, by either a physician or nurse. Further details are outlined in previous publications.^18,29^ Briefly, pre- and postbronchodilator spirometry was performed following the guidelines of the American Thoracic Society^36,41^ using a portable MedGraphics PC-based USB with calibration using a standard 3L syringe performed before each session. The criteria for testing eligibility has been outlined in a previous publication.^29^ Participants were included if they had no acute respiratory symptoms for at least 3 weeks and were instructed to refrain from using asthma medications before testing; those with symptoms were rescheduled. Measurements were reported from a minimum of 3 (and no more than 8) maneuvers, including FEV_1_ (liters), FVC (liters), FEV_1_/FVC ratio, and forced expiratory flow between 25% and 75% of lung volume (FEF_25%–75%_, liters). Participants received two (200 mcg total) puffs of salbutamol through a spacer with mask and spirometry was repeated after 15 minutes. All test results underwent review by a pediatric pulmonologist to ensure acceptability and repeatability. Lung function parameters were converted into z-scores standardized for age, height, and sex, with a mean of 0 and a standard deviation of 1, using multivariable regression.^42^ The z-score of FEV_1_/FVC ratio was calculated by dividing observed values of FEV_1_ by FVC, divided by their standard deviation (SD).

### Covariates

We included covariates known to be associated with both metal exposure and childhood lung function. Figure S2; https://links.lww.com/EE/A393 shows the Directed Acyclic Graph used for variable selection. The covariates included were mother’s age (years) and maternal education at enrollment (categorized as less than high school, some high school or high school graduate, and more than high school); child’s sex (male/female); and environmental tobacco smoke exposure (yes/no), based on report of smoker in the home during the second or third trimesters of pregnancy. Asthma diagnosis was determined using the Spanish-validated International Study of Asthma and Allergies in Childhood questionnaire,^43^ based on the caregiver’s affirmative responses to the questions: “During the past year, has your child had episodes of wheezing or whistling in the chest?” and “Has your child ever been diagnosed with asthma in their life?.” Birth weight-for-gestational-age z-scores were calculated using the Fenton growth reference.^44^ Gestational age (weeks) was estimated using a combination of last menstrual period and the Capurro method.^45^ Preterm birth was defined as delivery before 37 weeks of gestation.

### Statistical analysis

Descriptive statistics were performed for the variables of interest, with results displayed as mean (SD), median (interquartile range) for continuous variables, or frequency (percentage) for categorical variables. The relationship between metal concentrations in the second and third trimesters was examined using Spearman correlation coefficients. To assess trimester-specific associations, analyses were performed separately for the second and third trimester.

We performed multiple linear regressions to evaluate the associations between individual second and third trimester blood metals and postbronchodilator lung function parameters stratified by sex. Estimates reflect the change in the mean z-score of lung function parameters per doubling of metal levels. We used a weighted quantile sum (WQS) regression model, as described previously,^46,47^ to assess the joint effects of the metals mixture on lung function parameters for each sex. This model produces a weighted index $\left(\Sigma_{i=1}^{c}w_{i}q_{i}\right)$, to estimate the mixture effect on an outcome, and identifies chemicals of concern; where *w*_i_, is the weight associated with the i^th^ component of the mixture averaged over 100 bootstraps, and q_i_ is the quantile of the i^th^ component.^46,48^ To assess sex-specific effects, we incorporated an interaction term, WQS × sex, and used weights calculated separately for each stratum (male/female).^49,50^ The relative weights were computed by dividing the average weight of each chemical by the total weight within the respective strata (e.g., males or females). This ensures that the relative weights within each stratum also sum up to 100%.^48^ We a priori determined to examine associations in the negative direction (inversely) associated with children’s lung function parameters. To obtain robust estimates, we included a repeated holdout validation technique, which involves repeating the WQS analysis across 100 holdouts, with the data randomly partitioned into 40/60 training and validation splits.^51^ This yielded a range of mean estimates and chemical weights, alongside their 95% confidence intervals (CIs).^51^ As outlined by Tanner et al,^51^ we considered an effect to be present when at least 97.5% of the effect estimates were either above or below the null value in a two-sided test. If the mean estimate suggested an association, we identified potentially influential chemicals using the “Busgang criteria,” as previously described, to assess the contribution of each metal to the observed mixture effect.^49,50^

To account for potential synergistic and nonlinear effects among metal mixture components, we fitted a sex-stratified hierarchical BKMR.^52^ Metals were grouped into two categories according to their roles in biological systems: essential metals (Co, Cu, Mn, Se, and Zn) and nonessential metals (As, Cd, Ni, and Pb)^53,54^ using hierarchical variable selection, running 50,000 iterations to estimate the posterior inclusion probabilities (PIPs) of highly correlated exposures. PIPs values range from 0 to 1 and quantify the relevance of each exposure, with a value of 1 indicating inclusion in all iterations. To determine important predictors, we used the standard cutoff of 0.5.^55^ Before BKMR analyses, metal concentrations were centered and scaled. We evaluated (1) linearity of associations using univariate exposure–response plots with 95% CI’s; (2) overall metal mixture associations, presented as changes in the estimated effect by a simultaneous quantile increase in all mixture components relative to their median concentrations. Graphical outputs were used to display these results. In sensitivity analysis, we additionally adjusted for Fenton birthweight z-score, gestational age, and preterm birth. Analyses were performed in R version 4.3.2 (R Foundation for Statistical Computing, Vienna, Austria) and Stata v 18 (Stata, College Station, TX). The WQS and BKMR analyses were conducted using the “gWQS” and “bkmr” packages in R.

## Results

### Study participant characteristics

Table 1 displays the sociodemographic, exposure, and outcome characteristics for the mother-child dyads for the overall sample and stratified by sex. The mean maternal age at enrollment was 27.63 years (SD: 5.54). Most of the mothers included in the study (40.0%) reported having 12 or fewer years of education, while 39.3% indicated prenatal exposure to environmental tobacco smoke. Approximately 54.6% of the children were male. Caregivers reported that 2.28% of the participating children had current asthma. The characteristics of children included in the study were comparable to those of age-eligible children who were not included (Table S1; https://links.lww.com/EE/A393). Spearman correlation coefficients between metals measured in second and third trimester ranged from weak to moderate (Figure S3; https://links.lww.com/EE/A393).

**TABLE 1.** Overall and Sex-Stratified Maternal and Child Characteristics of Included Participants in the PROGRESS Study

	Overall Sample N = 438	Males N = 239	Females N = 199
Categorical variables	n (%)		
Maternal education at enrollment			
Less than high school	175 (39.95)	105 (43.93)	70 (35.18)
High school	159 (36.30)	75 (31.38)	84 (42.21)
More than high school	104 (23.74)	59 (24.69)	45 (22.61)
Prenatal ETS exposure^a^			
Yes	172 (39.27)	102 (42.68)	70 (35.18)
No	266 (60.73)	137 (57.32)	129 (64.82)
Current child asthma			
Yes	10 (2.28)	7 (2.93)	3 (1.51)
No	428 (97.72)	232 (97.07)	196 (98.49)
Continuous variables			
Maternal age at enrollment (years) (mean, SD)	27.6 (5.5)	28.0 (5.4)	27.2 (5.7)
Child age at spirometry (mean, SD)	10.81 (1.85)	10.93 (1.82)	10.66 (1.87)
Child height at spirometry (cm) (mean, SD)	145 (12.9)	147 (13.8)	143 (11.5)
Second-trimester blood metal concentrations (μg/L) (median, IQR)			
Arsenic	0.73 (0.33)	0.73(0.33)	0.74 (0.33)
Cadmium	0.23(0.16)	0.25 (0.16)	0.23 (0.17)
Cobalt	0.17 (0.12)	0.16 (0.13)	0.18 (0.12)
Copper	1540.35(308.23)	1547.49 (309.66)	1530.86 (294.70)
Manganese	13.92 (5.84)	14.16 (5.35)	13.59 (6.16)
Nickel	2.52 (3.52)	2.49(3.88)	2.58 (3.45)
Lead	28.22 (24.76)	29.88(26.29)	26.60 (22.46)
Selenium	242.45 (43.57)	242.86 (45.24)	240.46 (41.04)
Zinc	5943.93 (1246.83)	5934.74 (1189.90)	5961.18 (1300.06)
Third-trimester blood metal concentrations (μg/L; median, IQR)			
Arsenic	0.73 (0.41)	0.76 (0.44)	0.70 (0.40)
Cadmium	0.22 (0.15)	0.24 (0.15)	0.22 (0.14)
Cobalt	0.26 (0.17)	0.26 (0.18)	0.27 (0.17)
Copper	1552.49 (301.92)	1579.70 (314.44)	1539.31 (254.58)
Manganese	18.27 (7.23)	17.78 (7.46)	18.67 (6.90)
Nickel	2.35 (2.29)	2.37 (2.19)	2.27 (2.47)
Lead	29.61 (28.14)	30.80 (29.01)	27.17 (25.78)
Selenium	237.06 (33.84)	239.88 (35.45)	231.46 (32.01)
Zinc	6313.31 (1208.10)	6327.29 (1225.42)	6296.09 (1195.71)
Respiratory outcomes (median, IQR)			
Z-score of FEV_1_^b^	−0.01 (1.10)	−0.02 (1.21)	0.00 (1.08)
Z-score of FVC^b^	0.03 (1.09)	0.01 (1.11)	0.04 (1.06)
Z-score of FEF_25%–75%_^b^	−0.03 (1.15)	−0.03 (1.09)	−0.05 (1.25)
FEV_1_/FVC ratio^b^	−0.03 (1.15)	−0.03 (1.09)	−0.05 (1.25)

a Maternal report of smokers inside the home at second or third trimester of pregnancy.

b Adjusted for age, sex, and height.

FEV_1_ indicates forced expiratory volume in 1 second; FVC, forced vital capacity; FEF_25%–75%_, forced expiratory flow at 25%–75% of the pulmonary volume; ETS, environmental tobacco smoke; IQR, interquartile range.

### Individual associations of metals concentrations and lung function

**Figure 1.**
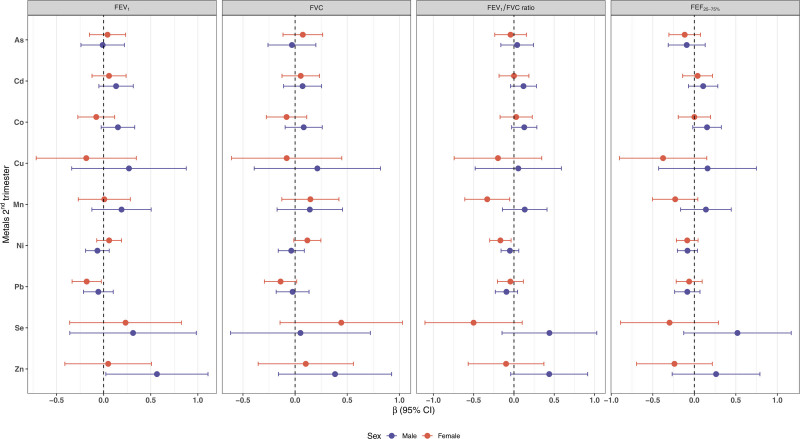
Adjusted associations from multiple linear regressions between each log_2_-transformed metals of second trimester blood with children’s lung function tests. The models were adjusted for maternal age, education, and ETS. ETS indicates environmental tobacco smoke; FEV_1_, forced expiratory volume in 1 second; FVC, forced vital capacity; FEF_25%−75%_, forced expiratory flow between 25% and 75%.

We observed that, in the overall sample, each doubling of second trimester Ni exposure was associated with a lower FEV_1_/FVC ratio (β = −0.11, 95% CI: −0.19, −0.02) (Table S2; https://links.lww.com/EE/A393). A doubling of Pb concentration in second trimester in females was associated with lower FEV_1_ z-scores (β = −0.18, 95% CI: −0.34, −0.02) (Figure 1), with negative and null association in males (β = −0.06, 95% CI: −0.21, 0.10). Additionally, a doubling of Zn concentration in second trimester was associated with increased FEV_1_ z-scores in males (β = 0.57, 95% CI: 0.02, 1.11), whereas in females estimates were null (β = 0.05, 95% CI: −0.41, 0.51). In females, second trimester Mn concentration was associated with lower FEV_1_/FVC ratio (β = −0.33, 95% CI: −0.61, −0.05), whereas in males there was a nonsignificant positive association (β = 0.13, 95% CI: −0.14, 0.41). A doubling of second trimester Ni concentration was associated with lower FEV_1_/FVC ratio (β = −0.17, 95% CI: −0.3, −0.04) in females, with a weaker negative association in males (β = −0.05, 95% CI: −0.16, 0.06).

**Figure 2.**
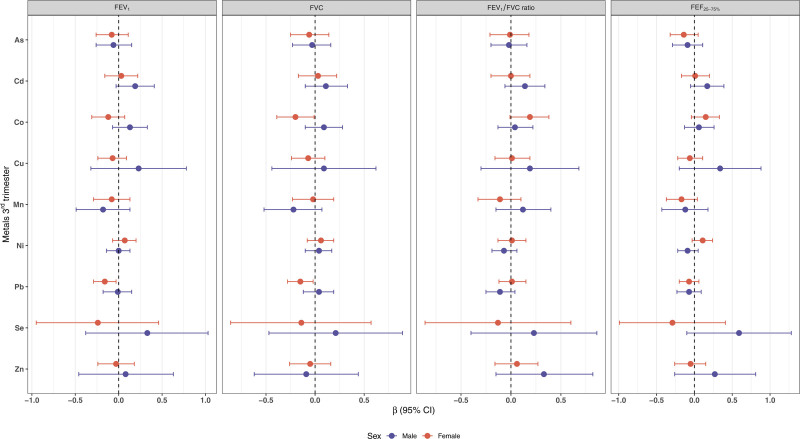
Adjusted associations from multiple linear regressions between each log_2_-transformed metals of third trimester blood with children’s lung function tests. The models were adjusted for maternal age, education, and ETS. ETS indicates environmental tobacco smoke; FEV_1_, forced expiratory volume in 1 second; FVC, forced vital capacity; FEF_25%−75%_, forced expiratory flow between 25% and 75%.

Regarding third trimester exposure, null associations were observed between individual metals and lung function parameters in the overall sample (Table S3; https://links.lww.com/EE/A393) while sex-stratified analyses revealed inverse associations among females (Figure 2). Specifically, each doubling of Pb concentration was associated with lower FEV_1_ z-scores (β = −0.16, 95% CI: −0.29, −0.03) and a null association in males (β = −0.01, 95% CI: −0.18, 0.15). Similarly, increased concentration of Co (β = −0.20, 95% CI: −0.39, −0.01) and Pb (β = −0.15, 95% CI: −0.28, −0.02) were each associated with lower FVC z-scores in females, while in males, positive and null associations were observed (β = 0.09, 95% CI: −0.10, 0.28; β = 0.04, −0.12, 0.19, respectively).

### Overall associations of metals mixture and lung function

In the overall sample, an association between higher concentrations of second trimester metals mixture and reduced FEV_1_/FVC ratio −0.15 (95% CI: −0.28, −0.03) was observed. The possible chemicals of concern were Ni, Pb, and Mn. We did not observe any other associations between second and third trimester blood metal mixtures and FEV_1_, FVC, and FEF_25%–75%_ z-scores (Table S4; https://links.lww.com/EE/A393).

### Sex-specific associations of metals mixture and lung function

**Figure 3.**
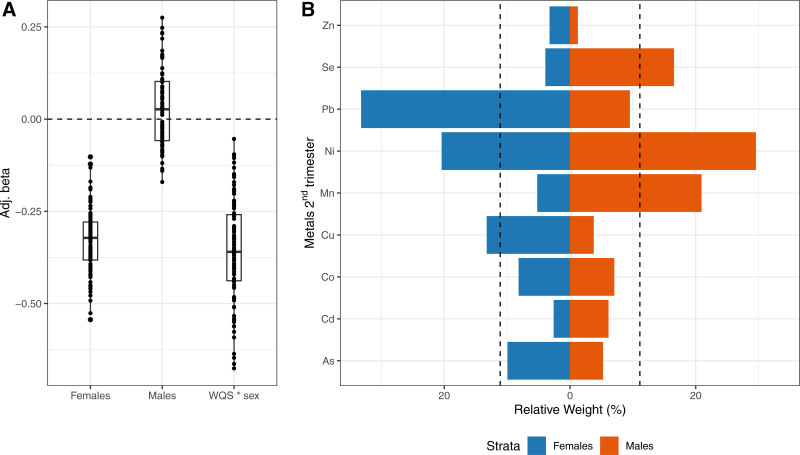
Mean adjusted betas (A) and sex-specific relative weights (B) from a WQS (negative constraint) linear regression with 100 repeated holdouts examining associations between second trimester metals mixtures and FEV_1_/FVC ratio. The model was adjusted for maternal age, education, and ETS. Panel (A) shows beta estimates across holdouts; each dot represents one iteration. Panel (B) shows average relative weights for each chemical by sex (males/females). Dotted line indicates the threshold (11.11%) for metals of concern. ETS indicates environmental tobacco smoke; FEV_1_, forced expiratory volume in 1 second; FVC, forced vital capacity.

**Figure 4.**
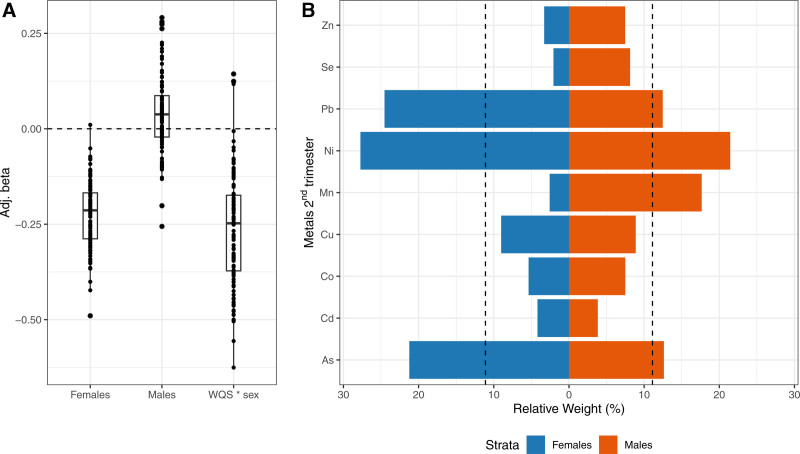
Mean adjusted betas (A) and sex-specific relative weights (B) from a WQS (negative constraint) linear regression with 100 repeated holdouts examining associations between second trimester metals mixtures and FEF_25%–75%_ z-score. The model was adjusted for maternal age, education, and ETS. Panel (A) shows beta estimates across holdouts; each dot represents one iteration. Panel (B) shows average relative weights for each chemical by sex (males/females). Dotted line indicates the threshold (11.11%) for metals of concern. ETS indicates environmental tobacco smoke; FEF_25%−75%_, forced expiratory flow between 25% and 75%.

In sex-stratified WQS models, we found an inverse association between second trimester metals mixture and lower FEV_1_/FVC ratio in females (mean β = −0.33, 95% CI: −0.49, −0.15), and a null positive association in males (mean β = 0.03, 95% CI: −0.13, 0.23). Pb, Ni, and Cu were the primary contributors to the mixture effect in females (Figure 3). Similarly, we observed an inverse association between prenatal metal mixtures during the second trimester and FEF_25%–75%_ z-scores in females (mean β = −0.22, 95% CI: −0.38, −0.07) compared with males (mean β = 0.04, 95% CI: −0.13, 0.28). Pb, Ni, and As were the top contributors to the mixture effect seen in females (Figure 4). Additional adjustment for birthweight z-score, gestational age, and preterm birth did not modify these results (Figures S6 and S7; https://links.lww.com/EE/A393). We did not find evidence of additional sex-specific associations for second and third trimesters metals mixture exposure and lung function parameters in childhood (Figures S4, S5, and S8–S11; https://links.lww.com/EE/A393).

**Figure 5.**
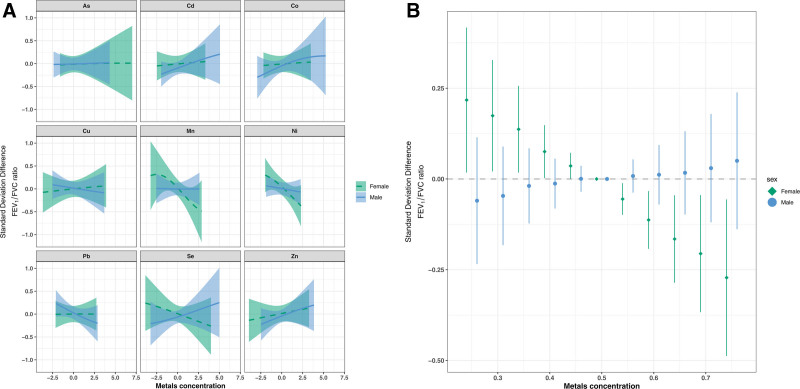
Hierarchical BKMR mixture sex-specific associations between second trimester metals with FEV_1_/FVC ratio. The model was adjusted for maternal age, education, and ETS. Univariate exposure–response plots with 95% credible intervals (A) and overall mixture association with FEV_1_/FVC ratio (B). ETS indicates environmental tobacco smoke; FEV_1_, forced expiratory volume in 1 second; FVC, forced vital capacity.

Sex-stratified BKMR analyses yielded group and conditional PIPs for lung function parameters across two trimesters and are shown in Tables S5 and S6; https://links.lww.com/EE/A393. Evidence of a metal mixture association at second trimester with FEV_1_/FVC ratio was observed in females (Figure 5), and the highest conditional PIPs in the female stratum were for Ni and Mn (Table S5; https://links.lww.com/EE/A393). Additional adjustment for birthweight z-score, gestational age, and preterm birth did not modify this finding (Figure S12; https://links.lww.com/EE/A393). Additional models showed no evidence of any other relevant associations (Figures S13–S19; https://links.lww.com/EE/A393).

## Discussion

This study aimed to evaluate associations between prenatal exposure to metals and childhood lung function, including potential sex-specific effects. We found that associations between individual metals and lung function parameters differed by sex using multiple linear regression models. Additionally, higher exposure to a metals mixture during the second trimester was associated with lower FEV_1_/FVC ratio and FEF_25%–75%_ z-score in a sex-dependent manner, as identified using supervised mixture methods.

Our results are in line with previous work identifying As, Pb, Mn, Co, Cu, and Ni as metals affecting lung function. Children in communities from a north-central part of Mexico who were exposed *in utero* and in early childhood to As showed decreased FVC values (β = −0.003, 95% CI: −0.006, −0.001) and a restrictive spirometric pattern in 57% of the study sample.^56^ Signes-Pastor et al^13^ reported that a two-fold increase in maternal Co and Pb was associated with a decline in FVC z-score in multiple linear regression models. In our study, although CIs overlapped across sex strata and thus do not provide strong evidence of interaction from individual-metal regression analyses, we observed inverse associations of Pb and Co with lung function parameters among females during the third trimester. These findings align with those of Signes-Pastor et al., who also identified late-gestation windows (24–28 weeks) as critical periods of susceptibility for these associations in the New Hampshire Birth Cohort Study. Similarly, Madrigal et al^57^ reported an inverse association between urinary Mn and the FEV_1_/FVC ratio among children in the United States, which is consistent with the negative associations we observed for second trimester Mn exposure among Mexican females. Furthermore, the effect of urinary Mn was significantly inversely correlated with predicted FVC% (β = −1.463), FEV_1_% (β = −2.235), and FEV_1_/FVC % (β = −0.983) in young adult females from the YOung TAiwanese Cohort Study.^58^ In a subsample of 39 children with diagnosed asthma in Chicago, Illinois, higher toenail Cu levels were associated with lower values of natural log-transformed FEV_1_, FEV_1_/FVC ratio, and FEF_25%–75%_.^59^ Evidence from children aged 10 years living in an e-waste recycling town in China revealed that those exposed to high levels of Ni had lower FVC values. In contrast, older age groups showed no differences compared to children from a control area, suggesting that as children grow and their respiratory systems mature, their ability to repair tissue damage and remove waste may improve, which might explain why this inverse association is no longer seen in older age groups.^60^ This supports the possibility that the impact of exposure to metals may vary across different stages of childhood and adolescence.

Prenatal exposure to nonessential metals, such as Pb, As, and Cd, can impact lung growth and development by inducing oxidative stress, damage to epithelial cells, and lung inflammation, which may contribute to the development of pediatric respiratory diseases, such as asthma.^13,29,61^ Similarly, Ni compounds may induce inflammatory responses in alveoli and a type 1 hypersensitivity reaction, which may lower FEV_1_ and FEF_25%–75%_ values.^62^ Additionally, essential metals have adverse effects. Exposure to Mn has been associated with increased production of inflammatory cytokines in human bronchial epithelial cells and vascular endothelial growth factor production.^57,63,64^ Moreover, exposure to Co and Cu has been implicated in impaired lung function in production workers, as they can enhance the production of reactive oxygen species.^13,65,66^ However, group PIPs for nonessential metals in BKRM analyses tended to be higher compared to essential metals, suggesting potential differential relevance in their contribution to lung function, even in the absence of a significant overall mixture effect.

Prenatal exposure to metals may influence childhood lung function with effects that differ by sex. Metals can act as endocrine-disrupting chemicals by interfering with sex steroids.^67,68^ Estrogens and androgens influence lung development and function, as their receptors are expressed in the fetal lung and contribute to sex-specific differentiation. In humans, females exhibit earlier surfactant production and maturation,^34,69–71^ which may partly underlie the higher susceptibility of males to respiratory diseases. However, estrogen appears to play a major role in pulmonary inflammatory processes by regulating the synthesis of cytokines and inflammatory mediators.^72^ Exposure to metals has also been shown to affect immune regulation through alterations in immune cell generation, shifts in inflammatory markers, and cytokine dysregulation.^73^ Importantly, both the developmental window of exposure and sex are critical factors in determining the immunotoxic effects of metals such as Pb and Cd during early life.^17^ Epigenetic mechanisms may also underlie sex-specific effects of prenatal metal exposure on lung function by linking environmental exposures with genetic factors.^74,75^ *In utero* exposure to multiple metals has been associated with differential DNA methylation in cord blood, with some effects persisting into mid-childhood,^76^ and methylation of DNA at later ages has been related to lung function parameters, including sex-specific associations.^77^ Lung development has critical stages, notably during the second and third trimesters, aligning with the pseudoglandular and canalicular stages.^2,4^ We identified sex-specific interactions during these trimesters, which may underlie the significance of metal exposure, impacting lung development differently based on timing and sex.

Although the literature often reports male offspring as more vulnerable to impaired lung development.^30,70^ Our findings indicated stronger associations among females. Females may experience higher internal doses of certain metals due to sex-specific differences in absorption and metabolism, as reported for Mn.^58,78^ Additionally, hormonal and epigenetic modulation could enhance the susceptibility of females to inflammatory or oxidative stress responses following metal exposure. Lower FEV_1_/FVC ratio and FEF_25%–75%_ z-score, observed in mixture analyses among females, may reflect airflow obstruction.^59^ This interpretation is consistent with findings from the PRISM cohort, where prenatal exposure to metal mixtures was associated with increased total airway resistance, an effect more pronounced among females.^79^ Together, these results suggest that prenatal exposure to metal mixtures may impair airway function in a sex-specific manner.

Our study has several strengths. The PROGRESS study is a prospective birth cohort with high-quality post-bronchodilator spirometric values along with comprehensive information on environmental factors and sociodemographic characteristics. We were able to examine metals exposure at two different timepoints during pregnancy. In environmental epidemiology, generalized linear regression models offer a straightforward approach to evaluate associations between individual chemicals and health outcomes.^80^ In contrast, applying both WQS and BKMR models provides complementary insights to evaluate the sex-specific effects of metal mixtures on lung function. Together, these three modeling approaches capture distinct aspects, and their joint interpretation offers a more nuanced understanding of sex-specific effects. We also acknowledge certain limitations. Associations in our study were limited to second trimester blood metal mixtures, whereas prior work using dentin suggested effects during late gestation (late second to early third trimester of pregnancy). This discrepancy may reflect differences in biomarker properties. Although blood samples are advantageous for the long-term assessment of certain metals, such as Pb, others, such as As, are rapidly cleared, making blood a less suitable biomarker of exposure in those cases,^81^ which could result in measurement error. In contrast, teeth integrate fetal uptake at weekly resolution, enabling the identification of critical windows of susceptibility. It should be noted that in the aforementioned study, metal dentin measurements spanned from 15 before 15 weeks after birth, meaning the second trimester was not entirely covered in this analysis.^18^ A limitation of our study is the lack of exposure data during the first trimester. PROGRESS recruited women starting in the second trimester of pregnancy, therefore data on metal concentrations is only available for the second and third trimesters of pregnancy. However, our exposure assessment does align with critical stages in lung development, the pseudoglandular and canalicular stages,^2,4^ as stated in the discussion section. Nonetheless, we cannot rule out the impact of these exposures at other times points in the early prenatal and postnatal period. Our sample size may have hampered our ability to identify more sex-specific associations. We also aim to follow up our participants to see if these associations persist.

In summary, these findings suggest that females may be more vulnerable to the effects of prenatal exposure to metal mixtures on lung function. This work underscores the need to understand critical windows of exposure and sex-specific differences. Additional evidence on widespread environmental chemicals can guide strategies aimed at reducing risk and addressing factors that may influence lifelong respiratory and nonrespiratory morbidity.

## Acknowledgements

We are grateful to the PROGRESS participants and staff at the National Institute of Public Health/Ministry of Health of Mexico and the National Institute of Perinatology. We thank the ABC (American British Cowdray Medical Center) in Mexico for providing some of the needed research facilities.

## Conflict of interest statement

The authors declare that they have no conflicts of interest with regard to the content of this report.

## Supplementary Material


