# Supplementary material for: Prenatal exposure to metal(loid)s mixture and childhood lung function: Exploring sex-specific associations
Source: Environ Epidemiol. 2025 Dec 3;9(6):e447. doi: 10.1097/EE9.0000000000000447 (PMC12677884; doi:10.1097/EE9.0000000000000447)

Supplemental material

Table S1. Characteristics of invited children compared with other age-eligible participants.

Table S2. Adjusted[a] associations from linear regressions between each $\log_2$(metals) of 2nd trimester blood with children's lung function tests.

Table S3. Adjusted[a] associations from linear regressions between each $\log_2$(metals) of 3rd trimester blood with children's lung function tests.

Table S4. Mean adjusted associations from WQS (negative constraint) linear regression models with 100 repeated holdouts between 2nd and 3rd trimester metals mixture and children's lung function**.**

Table S5. Group and conditional Posterior Inclusion Probabilities (PIPs) from the Bayesian kernel machine regression models evaluating associations between metals (nonessential/essential) in the 2nd and 3rd trimesters and childhood lung function.

Table S6. Group and conditional Posterior Inclusion Probabilities (PIPs) from the Bayesian kernel machine regression models evaluating associations between metals (nonessential/essential) in the 2nd and 3rd trimesters and childhood lung function.

Figure S1. Flow diagram of participants included in the analysis.

Figure S2. Directed Acyclic Graph (DAG) of assumed dependencies between prenatal exposure to metals, childhood lung function, and other socioeconomic and health related factors.

Figure S3. Correlation matrix (Spearman's ρ) of trimester specific metals among the study population.

Figure S4. Mean adjusted betas (A) and sex-specific relative weights (B) from a WQS (negative constraint) linear regression with 100 repeated holdouts examining associations between 2nd trimester metals mixtures and FEV_1 z-score.

Figure S5. Mean adjusted betas (A) and sex-specific relative weights (B) from a WQS (negative constraint) linear regression with 100 repeated holdouts examining associations between 2nd trimester metals mixtures and FVC z-score.

Figure S6. Mean adjusted betas (A) and sex-specific relative weights (B) from a WQS (negative constraint) linear regression with 100 repeated holdouts examining associations between 2nd trimester metals mixtures and FEV_1/FVC ratio.

Figure S7. Mean adjusted betas (A) and sex-specific relative weights (B) from a WQS (negative constraint) linear regression with 100 repeated holdouts examining associations between 2nd trimester metals mixtures and $FEF_{25-75\%}$ z-score.

Figure S8. Mean adjusted betas (A) and sex-specific relative weights (B) from a WQS (negative constraint) linear regression with 100 repeated holdouts examining associations between 3rd trimester metals mixtures and FEV_1 z-score.

Figure S9. Mean adjusted betas (A) and sex-specific relative weights (B) from a WQS (negative constraint) linear regression with 100 repeated holdouts examining associations between 3rd trimester metals mixtures and FVC z-score.

Figure S10. Mean adjusted betas (A) and sex-specific relative weights (B) from a WQS (negative constraint) linear regression with 100 repeated holdouts examining associations between 3rd trimester metals mixtures and FEV_1/FVC ratio.

Figure S11. Mean adjusted betas (A) and sex-specific relative weights (B) from a WQS (negative constraint) linear regression with 100 repeated holdouts examining associations between 3rd trimester metals mixtures and $FEF_{25-75\%}$ z-score.

Figure S12. Hierarchical BKMR mixture sex-specific associations between 2nd trimester metals with FEV_1/FCV ratio.

Figure S13. Hierarchical BKMR mixture sex-specific associations between 2nd trimester metals with FEV_1 z-score.

Figure S14. Hierarchical BKMR mixture sex-specific associations between 2nd trimester metals with FVC z-score.

Figure S15. Hierarchical BKMR mixture sex-specific associations between 2nd trimester metals with $FEF_{25-75\%}$ z-score.

Figure S16. Hierarchical BKMR mixture sex-specific associations between 3rd trimester metals with FEV_1 z-score.

Figure S17. Hierarchical BKMR mixture sex-specific associations between 3rd trimester metals with FVC z-score.

Figure S18. Hierarchical BKMR mixture sex-specific associations between 3rd trimester metals with FEV_1/FVC ratio.

Figure S19. Hierarchical BKMR mixture sex-specific associations between 3rd trimester metals with $FEF_{25-75\%}$ z-score.

Table S1. Characteristics of invited children compared with other age-eligible participants.

	Excluded	Included
N (%)	509 (53.7)	438 (46.3)
Child's sex, n (%)		
Male	259 (50.88)	239 (54.57)
Female	250 (49.12)	199 (45.43)
Education, n (%)		
<Highschool	209 (41.06)	175 (39.95)
Highschool	175 (34.38)	159 (36.30)
>Highschool	125 (24.56)	104 (23.74)
Prenatal ETS exposure[a], n (%)		
Yes	176 (43.35)	172 (39.27)
No	230 (56.65)	266 (60.73)
Child asthma, n (%)		
Yes	0 (0)	10 (2.28)
No	509 (100)	428 (97.72)
Maternal age at enrollment (years) (mean, SD)	27.69 (5.44)	27.63 (5.54)
Second Trimester Blood Metal Concentrations (µg/L) (median, IQR)		
Arsenic	0.73 (0.34)	0.73 (0.33)
Cadmium	0.24 (0.17)	0.23 (0.16)
Cobalt	0.15 (0.11)	0.17 (0.12)
Copper	1,539.22 (279.96)	1,540.35 (308.23)
Manganese	13.52 (5.96)	13.92 (5.84)
Nickel	2.81 (3.64)	2.52 (3.52)
Lead	28.01 (24.80)	28.22 (24.76)
Selenium	244.83 (44.57)	242.45 (43.57)
Zinc	5,927.40 (1,348.11)	5,943.93 (1,246.83)
Third Trimester Blood Metal Concentrations (µg/L) (median, IQR)		
Arsenic	0.76 (0.40)	0.73 (0.41)
Cadmium	0.23 (0.18)	0.22 (0.15)
Cobalt	0.24 (0.18)	0.26 (0.17)
Copper	1,540.65 (310.17)	1,552.49 (301.92)
Manganese	18.37 (8.07)	18.27 (7.23)
Nickel	2.57 (2.63)	2.35 (2.29)
Lead	29.22 (25.66)	29.61 (28.14)
Selenium	237.35 (39.97)	237.06 (33.84)
Zinc	6,407.80 (1,276.17)	6,313.31 (1,208.10)

[a]Maternal report of smokers inside the home at second or third trimester of pregnancy.

Abbreviations: FEV_1, forced expiratory volume in one second; FVC, forced vital capacity; $FEF_{25\text{-}75\%}$, forced expiratory flow at 25-75% of the pulmonary volume.

Table S2. Adjusted[a] associations from linear regressions between each log_2(metals) of 2nd trimester blood with children's lung function tests.

Lung function parameter	Analyte	Overall			Males			Females		
		β	95% CI		β	95% CI		β	95% CI	
FEV_1 z-score	As	0.02	-0.13	0.16	-0.01	-0.24	0.22	0.04	-0.15	0.23
	Cd	0.1	-0.03	0.23	0.13	-0.05	0.31	0.06	-0.12	0.24
	Co	0.06	-0.08	0.19	0.15	-0.03	0.33	-0.08	-0.27	0.12
	Cu	0.04	-0.37	0.44	0.27	-0.34	0.88	-0.18	-0.72	0.35
	Mn	0.09	-0.12	0.3	0.19	-0.12	0.5	0.01	-0.27	0.28
	Ni	-0.01	-0.1	0.08	-0.07	-0.19	0.06	0.06	-0.07	0.19
	Pb	-0.11	-0.22	0	-0.06	-0.21	0.1	-0.18	-0.34	-0.02
	Se	0.26	-0.17	0.7	0.31	-0.36	0.98	0.23	-0.36	0.83
	Zn	0.28	-0.07	0.63	0.57	0.02	1.11	0.05	-0.41	0.51
FVC z-score	As	0.03	-0.12	0.17	-0.03	-0.26	0.2	0.07	-0.12	0.26
	Cd	0.06	-0.06	0.19	0.07	-0.11	0.25	0.05	-0.13	0.23
	Co	0.02	-0.12	0.15	0.08	-0.1	0.26	-0.08	-0.28	0.11
	Cu	0.06	-0.34	0.46	0.21	-0.39	0.82	-0.08	-0.61	0.45
	Mn	0.14	-0.06	0.35	0.14	-0.17	0.45	0.15	-0.13	0.42
	Ni	0.03	-0.06	0.12	-0.04	-0.16	0.09	0.12	-0.01	0.25
	Pb	-0.08	-0.19	0.03	-0.02	-0.18	0.13	-0.14	-0.29	0.02
	Se	0.25	-0.19	0.68	0.05	-0.62	0.72	0.44	-0.14	1.03
	Zn	0.22	-0.12	0.57	0.38	-0.16	0.93	0.1	-0.35	0.56
FEV_1/FVC ratio	As	0	-0.14	0.14	0.04	-0.16	0.24	-0.04	-0.24	0.15
	Cd	0.05	-0.07	0.17	0.12	-0.04	0.28	0	-0.18	0.19
	Co	0.08	-0.04	0.21	0.13	-0.03	0.28	0.03	-0.17	0.23
	Cu	-0.1	-0.48	0.28	0.05	-0.48	0.59	-0.20	-0.74	0.34
	Mn	-0.12	-0.32	0.08	0.13	-0.14	0.41	-0.33	-0.61	-0.05
	Ni	-0.11	-0.19	-0.02	-0.05	-0.16	0.06	-0.17	-0.3	-0.04
	Pb	-0.08	-0.18	0.03	-0.09	-0.23	0.05	-0.04	-0.2	0.12
	Se	-0.09	-0.51	0.32	0.44	-0.15	1.03	-0.5	-1.1	0.1

	Zn	0.146	-0.189	0.48	0.44	-0.04	0.91	-0.1	-0.57	0.37
$FEF_{25-75\%}$ z-score	As	-0.096	-0.239	0.047	-0.09	-0.31	0.13	-0.12	-0.31	0.07
	Cd	0.083	-0.042	0.207	0.11	-0.07	0.28	0.04	-0.14	0.22
	Co	0.087	-0.04	0.215	0.15	-0.02	0.33	0	-0.19	0.2
	Cu	-0.117	-0.51	0.275	0.16	-0.43	0.75	-0.38	-0.90	0.15
	Mn	-0.062	-0.265	0.141	0.14	-0.16	0.44	-0.23	-0.5	0.04
	Ni	-0.081	-0.169	0.007	-0.08	-0.2	0.04	-0.08	-0.22	0.05
	Pb	-0.076	-0.184	0.032	-0.08	-0.24	0.07	-0.06	-0.22	0.09
	Se	0.08	-0.35	0.51	0.52	-0.13	1.17	-0.3	-0.89	0.29
	Zn	-0.003	-0.347	0.342	0.26	-0.27	0.79	-0.24	-0.69	0.22

[a]Adjusted for maternal age, maternal education at enrollment, and ETS. Abbreviations: CI, confidence interval; FEV1, forced expiratory volume in 1 s; FVC, forced vital capacity; FEF25−75%, forced expiratory flow between 25% and 75%; As, arsenic; Cd, cadmium; Co, cobalt; Cu, copper; Mn, manganese; Ni, nickel; Pb, lead; ETS, environmental tobacco smoke.

Table S3. Adjusted[a] associations from linear regressions between each log_2(metals) of 3rd trimester blood with children's lung function tests.

Lung function parameter	Analyte	Overall			Males			Females		
		β	95% CI		β	95% CI		β	95% CI	
FEV_1 z-score	As	-0.06	-0.2	0.07	-0.06	-0.26	0.15	-0.08	-0.26	0.11
	Cd	0.11	-0.03	0.26	0.19	-0.03	0.41	0.03	-0.16	0.22
	Co	0.01	-0.12	0.15	0.13	-0.07	0.33	-0.12	-0.31	0.07
	Cu	-0.04	-0.21	0.12	0.23	-0.32	0.78	-0.07	-0.24	0.09
	Mn	-0.12	-0.29	0.06	-0.18	-0.49	0.13	-0.08	-0.29	0.13
	Ni	0.03	-0.07	0.13	0	-0.14	0.13	0.07	-0.07	0.2
	Pb	-0.09	-0.19	0.007	-0.01	-0.18	0.15	-0.16	-0.29	-0.03
	Se	0.209	-0.28	0.693	0.33	-0.38	1.03	-0.24	-0.95	0.46
	Zn	-0.001	-0.2	0.196	0.08	-0.46	0.63	-0.03	-0.24	0.18
FVC z-score	As	-0.05	-0.18	0.086	-0.03	-0.23	0.16	-0.06	-0.25	0.14
	Cd	0.07	-0.07	0.21	0.11	-0.1	0.33	0.03	-0.17	0.22
	Co	-0.05	-0.18	0.089	0.09	-0.1	0.28	-0.2	-0.39	-0.01
	Cu	-0.06	-0.22	0.106	0.09	-0.44	0.62	-0.07	-0.24	0.1
	Mn	-0.1	-0.27	0.075	-0.22	-0.52	0.07	-0.02	-0.23	0.19
	Ni	0.04	-0.05	0.138	0.04	-0.1	0.17	0.06	-0.08	0.19
	Pb	-0.07	-0.17	0.03	0.04	-0.12	0.19	-0.15	-0.28	-0.02
	Se	0.21	-0.28	0.69	0.21	-0.47	0.89	-0.14	-0.86	0.57
	Zn	0	-0.2	0.2	-0.09	-0.62	0.44	-0.05	-0.26	0.16
FEV_1/FVC ratio	As	-0.04	-0.17	0.09	-0.02	-0.2	0.16	-0.01	-0.21	0.18
	Cd	0.06	-0.08	0.2	0.14	-0.06	0.34	0	-0.2	0.19
	Co	0.11	-0.02	0.24	0.04	-0.13	0.22	0.19	-0.01	0.38
	Cu	0.01	-0.15	0.17	0.19	-0.3	0.68	0.01	-0.16	0.19

	Mn	-0.04	-0.21	0.13	0.12	-0.15	0.4	-0.11	-0.33	0.1
	Ni	-0.04	-0.13	0.05	-0.07	-0.19	0.06	0.01	-0.13	0.15
	Pb	-0.06	-0.15	0.04	-0.11	-0.25	0.04	0.01	-0.12	0.15
	Se	-0.02	-0.49	0.45	0.23	-0.4	0.86	-0.13	-0.86	0.6
	Zn	0.08	-0.11	0.27	0.33	-0.15	0.82	0.06	-0.16	0.27
$FEF_{25-75\%}$ z-score	As	-0.11	-0.24	0.03	-0.09	-0.29	0.11	-0.14	-0.32	0.05
	Cd	0.1	-0.04	0.24	0.17	-0.05	0.39	0.01	-0.17	0.2
	Co	0.1	-0.04	0.23	0.06	-0.13	0.26	0.15	-0.04	0.33
	Cu	-0.02	-0.18	0.15	0.34	-0.2	0.88	-0.06	-0.22	0.11
	Mn	-0.15	-0.33	0.02	-0.12	-0.43	0.18	-0.17	-0.37	0.04
	Ni	0.01	-0.09	0.1	-0.09	-0.22	0.05	0.11	-0.03	0.24
	Pb	-0.07	-0.17	0.03	-0.07	-0.23	0.09	-0.07	-0.2	0.06
	Se	0.21	-0.28	0.69	0.59	-0.1	1.28	-0.29	-0.99	0.41
	Zn	0	-0.2	0.2	-0.27	-0.26	0.81	-0.05	-0.26	0.15

[a]Adjusted for maternal age, maternal education at enrollment, and ETS.

Abbreviations: CI, confidence interval; FEV1, forced expiratory volume in 1 s; FVC, forced vital capacity; FEF25−75%, forced expiratory flow between 25% and 75%; As, arsenic; Cd, cadmium; Co, cobalt; Cu, copper; Mn, manganese; Ni, nickel; Pb, lead; ETS, environmental tobacco smoke.

Table S4. Mean adjusted associations from WQS (negative constraint) linear regression models with 100 repeated holdouts between 2nd and 3rd trimester metals mixture and children's lung function.

**Lung function parameter[a]**	Estimate	SD-based 95%	
**2nd trimester mixture**			
FEV_1 z-score	0.01	-0.11	0.14
FVC z-score	0.10	-0.02	0.22
FEV_1/FVC ratio	-0.15	-0.28	-0.03
$FEF_{25-75\%}$ z-score	-0.09	-0.23	0.04
**3rd trimester mixture**			
FEV_1 z-score	-0.02	-0.15	0.12
FVC z-score	-0.00	-0.12	0.12
FEV_1/FVC ratio	0.04	-0.12	0.19
$FEF_{25-75\%}$ z-score	-0.03	-0.19	0.13

[a]The models were run with 100 repeated holdouts using 40% of the data as training and 60% as validation set. The models were adjusted for maternal age at delivery, education, and ETS. Abbreviations: FEV_1, forced expiratory volume in 1 s; FVC, forced vital capacity; $FEF_{25–75\%}$, forced expiratory flow between 25% and 75%; SD, standard deviation; ETS, environmental tobacco smoke.

Table S5. Group and conditional Posterior Inclusion Probabilities (PIPs) from the Bayesian kernel machine regression models evaluating associations between metals (nonessential/essential) in the 2nd and 3rd trimesters and childhood lung function.

Parameter	FEV_1 z-score				FVC z-score			
Sex	Males		Females		Males		Females	
Metals	Group PIP	Conditional PIP	Group PIP	Conditional PIP	Group PIP	Conditional PIP	Group PIP	Conditional PIP
2nd trimester								
Nonessential								
As	0.56	0.14	0.81	0.07	0.48	0.20	0.74	0.08
Cd		0.45		0.25		0.32		0.20
Ni		0.21		0.07		0.21		0.25
Pb		0.20		0.61		0.27		0.47
Essential								
Co	0.65	0.33	0.44	0.23	0.55	0.24	0.49	0.19
Cu		0.09		0.19		0.16		0.12
Mn		0.13		0.17		0.23		0.22
Se		0.08		0.23		0.11		0.35
Zn		0.37		0.18		0.25		0.12
3rd trimester								
Nonessential								
As	0.58	0.23	0.82	0.07	0.55	0.23	0.71	0.12
Cd		0.40		0.07		0.30		0.09
Ni		0.18		0.07		0.24		0.11
Pb		0.20		0.80		0.24		0.68
Essential								
Co	0.59	0.22	0.51	0.20	0.58	0.20	0.58	0.43
Cu		0.18		0.26		0.14		0.18
Mn		0.22		0.14		0.30		0.12
Se		0.18		0.14		0.15		0.09
Zn		0.20		0.26		0.21		0.17

Models were adjusted for maternal age at enrollment, maternal education, and ETS.
Abbreviations: FEV_1, forced expiratory volume in 1 s; FVC, forced vital capacity; $FEF_{25-75\%}$, forced expiratory flow between 25 and 75%; As, arsenic; Cd, cadmium; Ni, nickel; Pb, lead; Co, cobalt; Cu, copper; Mn, manganese; Se, selenium; Zn, zinc; ETS, environmental tobacco smoke.

Table S6. Group and conditional Posterior Inclusion Probabilities (PIPs) from the Bayesian kernel machine regression models evaluating associations between metals (nonessential/essential) in the 2nd and 3rd trimesters and childhood lung function.

Parameter	FEV_1/FVC ratio				$FEF_{25-75\%}$ z-score			
Sex	Males		Females		Males		Females	
Metals	Group PIP	Conditional PIP	Group PIP	Conditional PIP	Group PIP	Conditional PIP	Group PIP	Conditional PIP
2nd trimester								
Nonessential								
As	0.56	0.14	0.71	0.07	0.60	0.20	0.68	0.17
Cd		0.28		0.07		0.24		0.56
Ni		0.18		0.78		0.30		0.15
Pb		0.41		0.08		0.26		0.12
Essential								
Co	0.64	0.23	0.69	0.05	0.71	0.37	0.52	0.16
Cu		0.09		0.06		0.11		0.18
Mn		0.12		0.67		0.20		0.37
Se		0.27		0.15		0.20		0.12
Zn		0.29		0.06		0.12		0.17
3rd trimester								
Nonessential								
As	0.59	0.16	0.53	0.27	0.59	0.21	0.63	0.30
Cd		0.29		0.33		0.32		0.17
Ni		0.22		0.20		0.29		0.37
Pb		0.33		0.20		0.19		0.16
Essential								
Co	0.49	0.13	0.65	0.30	0.59	0.13	0.64	0.22
Cu		0.18		0.15		0.22		0.19
Mn		0.20		0.21		0.12		0.33
Se		0.18		0.12		0.34		0.11
Zn		0.31		0.22		0.19		0.15

Models were adjusted for maternal age at enrollment, maternal education, and ETS.
Abbreviations: FEV_1, forced expiratory volume in 1 s; FVC, forced vital capacity; $FEF_{25-75\%}$, forced expiratory flow between 25 and 75%; As, arsenic; Cd, cadmium; Ni, nickel; Pb, lead; Co, cobalt; Cu, copper; Mn, manganese; Se, selenium; Zn, zinc; ETS, environmental tobacco smoke.

Figure S1. Flow diagram of participants included in the analysis.

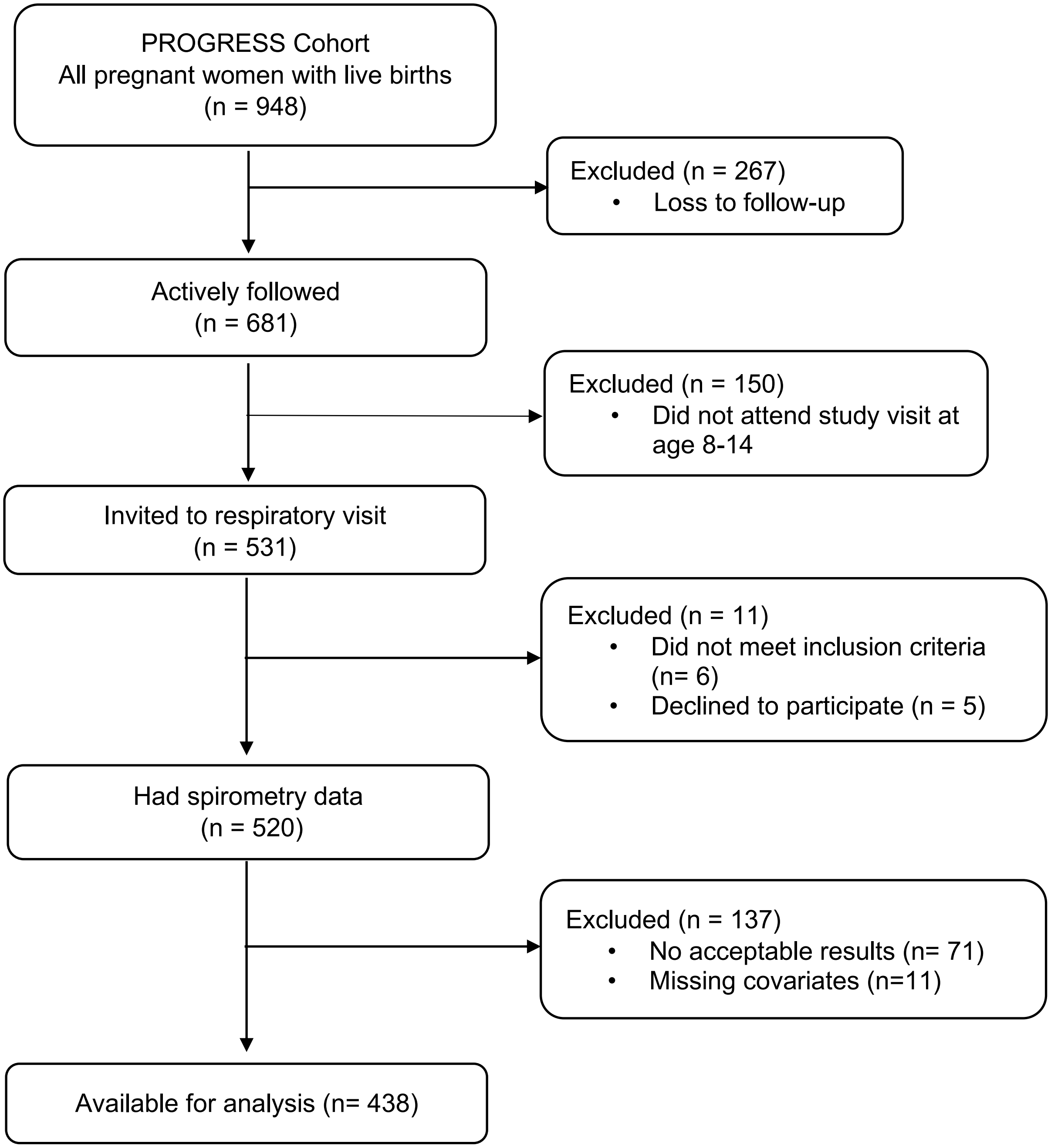

Figure S2. Directed Acyclic Graph (DAG) of assumed dependencies between prenatal exposure to metals, childhood lung function, and other socioeconomic and health related factors. Abbreviations: ETS, environmental tobacco smoke.

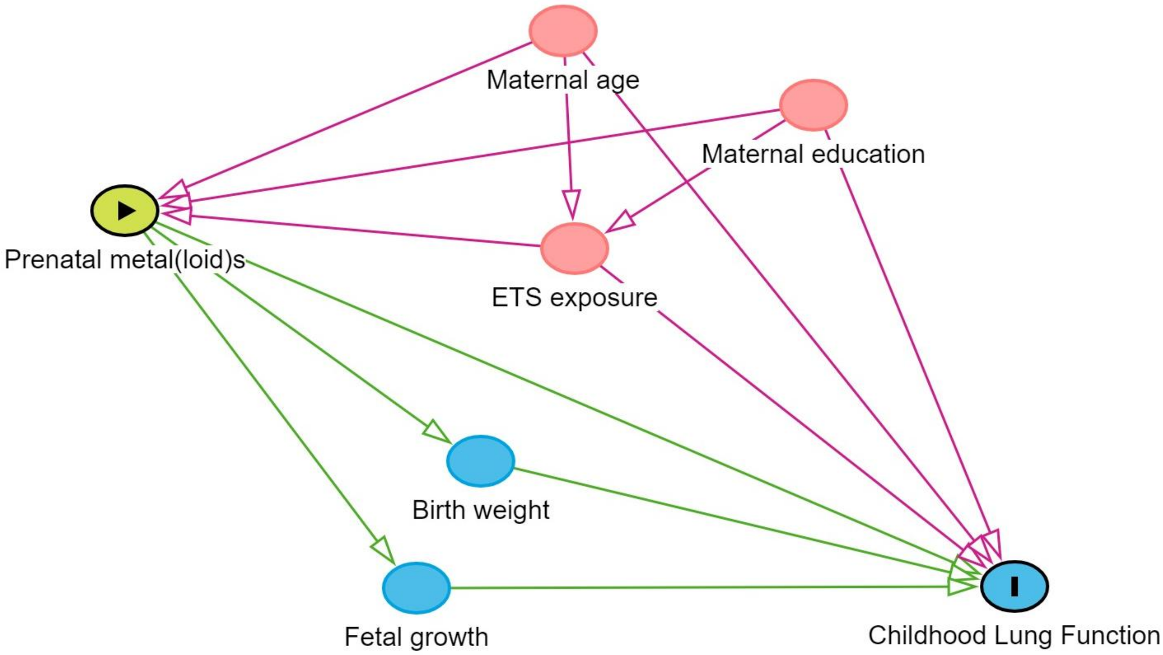

Figure S3. Correlation matrix (Spearman's ρ) of trimester specific metals among the study population. Abbreviations: As, arsenic; Cd, cadmium; Co, cobalt; Cu, copper; Mn, manganese; Ni, nickel; Pb, lead; Se, selenium; Zn, zinc.

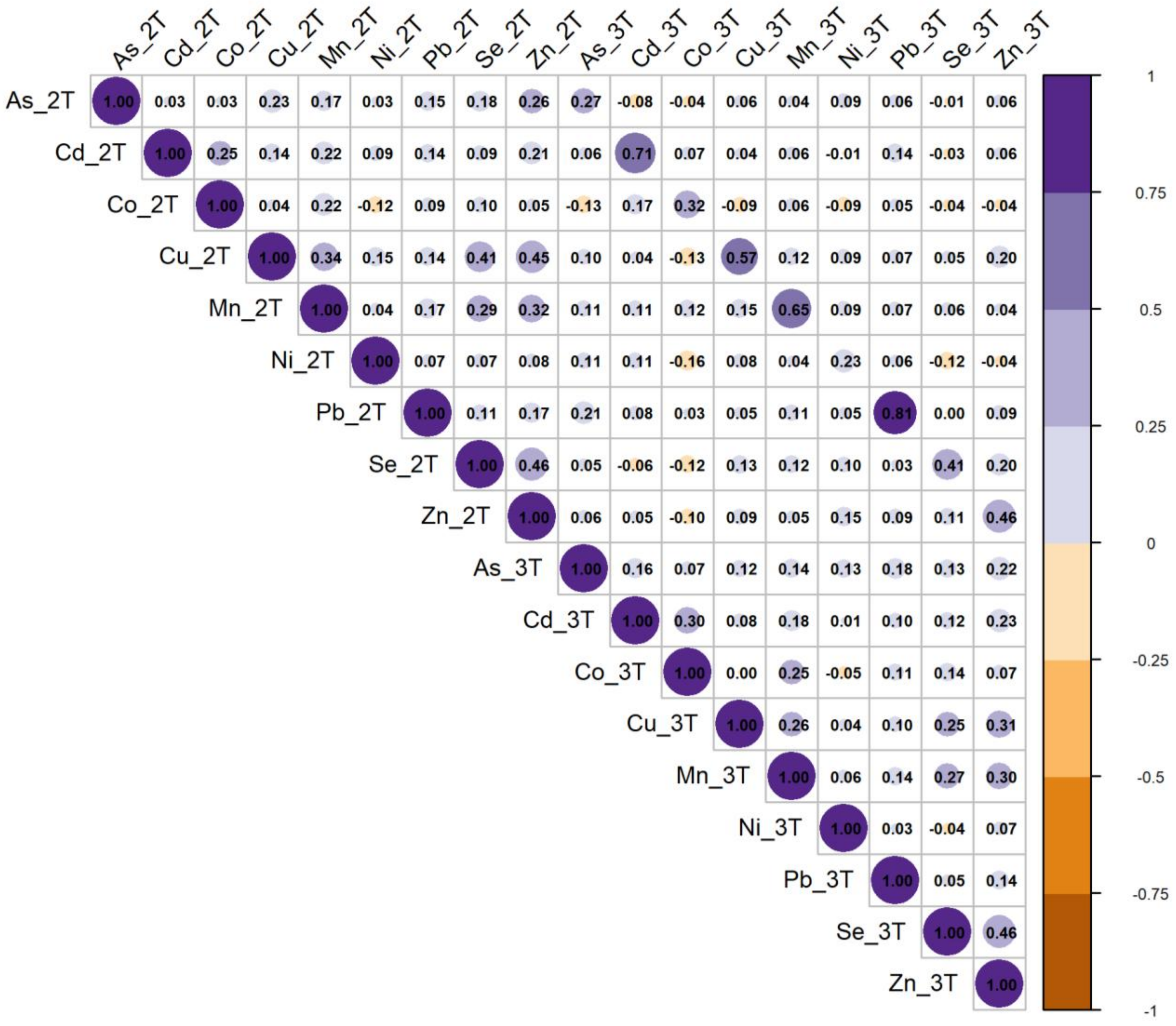

Figure S4. Mean adjusted betas (A) and sex-specific relative weights (B) from a WQS (negative constraint) linear regression with 100 repeated holdouts examining associations between 2nd trimester metals mixtures and FEV_1 z-score. The model was adjusted for maternal age, education, and ETS. Panel A shows beta estimates across holdouts; each dot represents one iteration. Panel B shows average relative weights for each chemical by sex (males/females). Dotted line indicates the threshold (11.11%) for metals of concern. Abbreviations: FEV_1, forced expiratory volume in 1 s; ETS, environmental tobacco smoke.

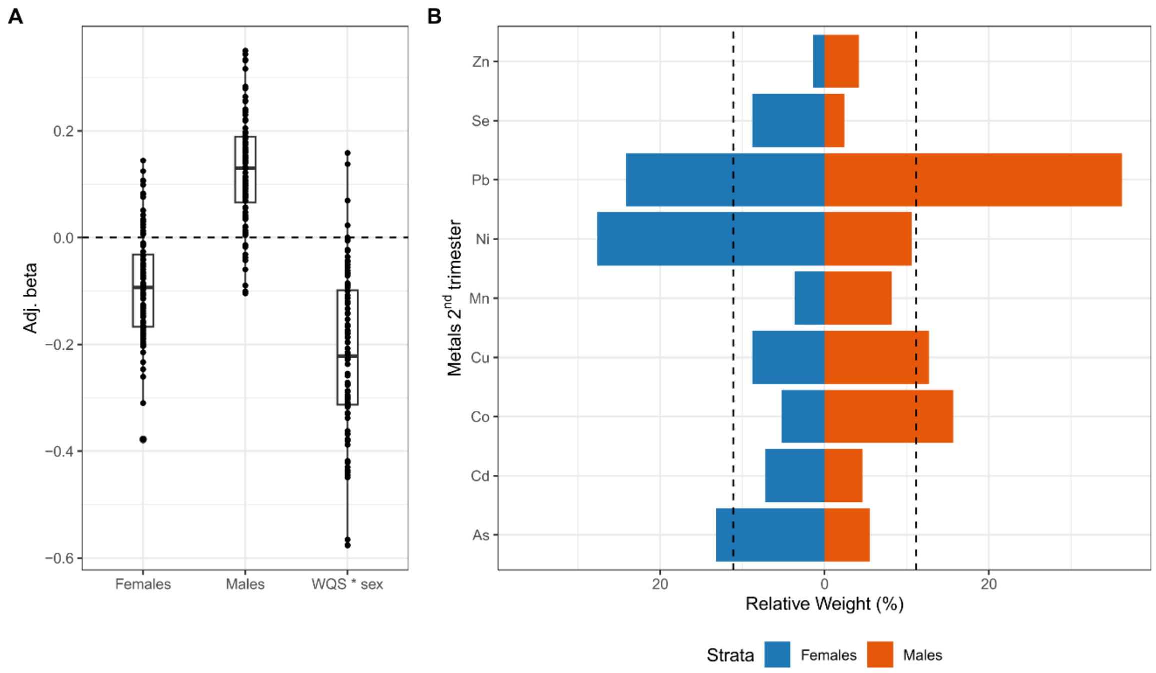

Figure S5. Mean adjusted betas (A) and sex-specific relative weights (B) from a WQS (negative constraint) linear regression with 100 repeated holdouts examining associations between 2nd trimester metals mixtures and FVC z-score. The model was adjusted for maternal age, education, and ETS. Panel A shows beta estimates across holdouts; each dot represents one iteration. Panel B shows average relative weights for each chemical by sex (males/females). Dotted line indicates the threshold (11.11%) for metals of concern. Abbreviations: FVC, forced vital capacity; ETS, environmental tobacco smoke.

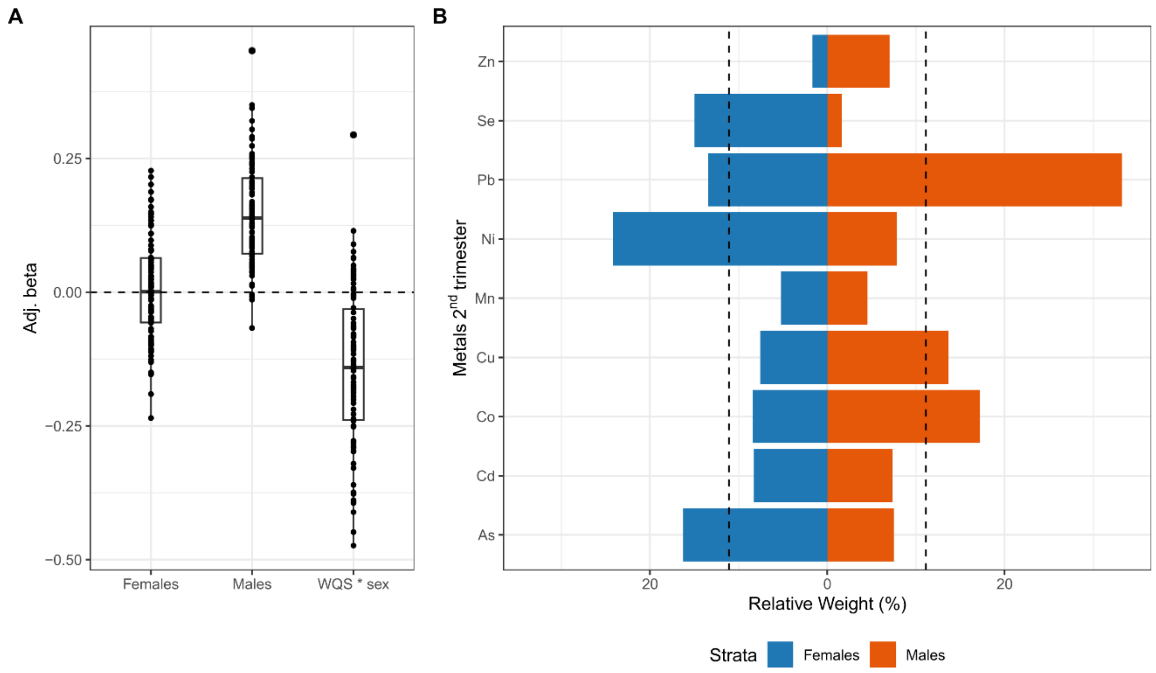

Figure S6. Mean adjusted betas (A) and sex-specific relative weights (B) from a WQS (negative constraint) linear regression with 100 repeated holdouts examining associations between 2nd trimester metals mixtures and FEV_1/FVC ratio. The model was adjusted for maternal age, education, ETS, birthweight z-score, gestational age, and preterm birth. Panel A shows beta estimates across holdouts; each dot represents one iteration. Panel B shows average relative weights for each chemical by sex (males/females). Dotted line indicates the threshold (11.11%) for metals of concern. Abbreviations: FEV_1, forced expiratory volume in 1 s; FVC, forced vital capacity; ETS, environmental tobacco smoke.

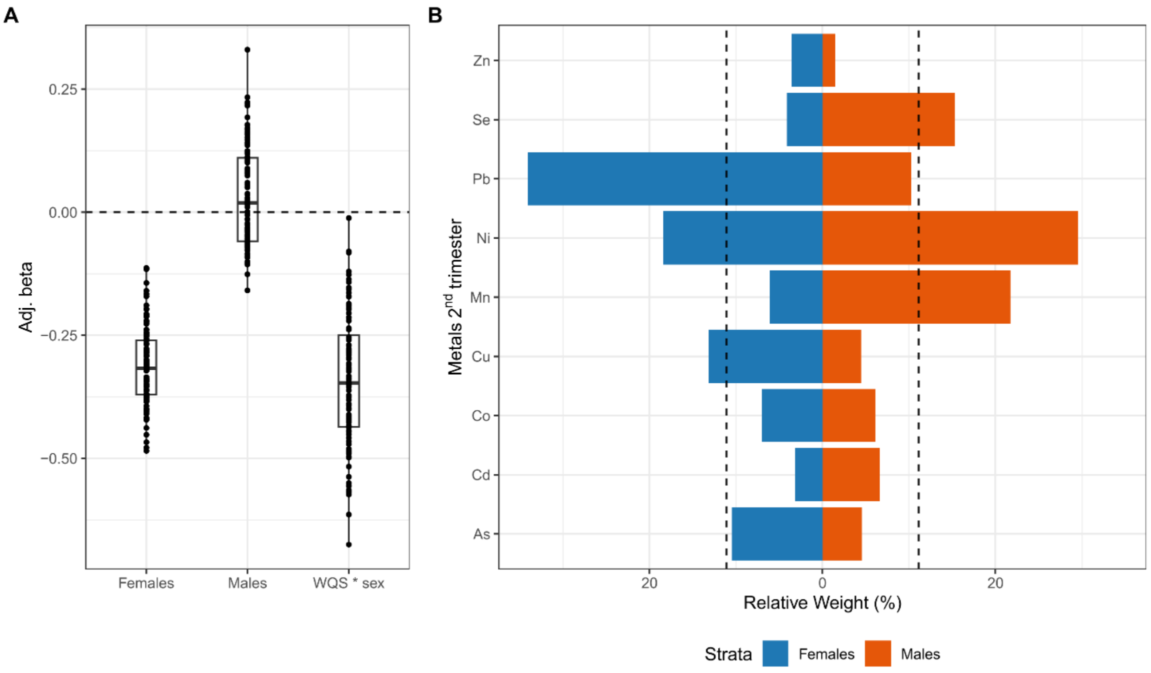

Figure S7. Mean adjusted betas (A) and sex-specific relative weights (B) from a WQS (negative constraint) linear regression with 100 repeated holdouts examining associations between 2nd trimester metals mixtures and $FEF_{25\text{-}75\%}$ z-score. The model was adjusted for maternal age, education, ETS, birthweight z-score, gestational age, and preterm birth. Panel A shows beta estimates across holdouts; each dot represents one iteration. Panel B shows average relative weights for each chemical by sex (males/females). Dotted line indicates the threshold (11.11%) for metals of concern. Abbreviations: $FEF_{25\text{–}75\%}$, forced expiratory flow between 25% and 75%; FVC, forced vital capacity; ETS, environmental tobacco smoke.

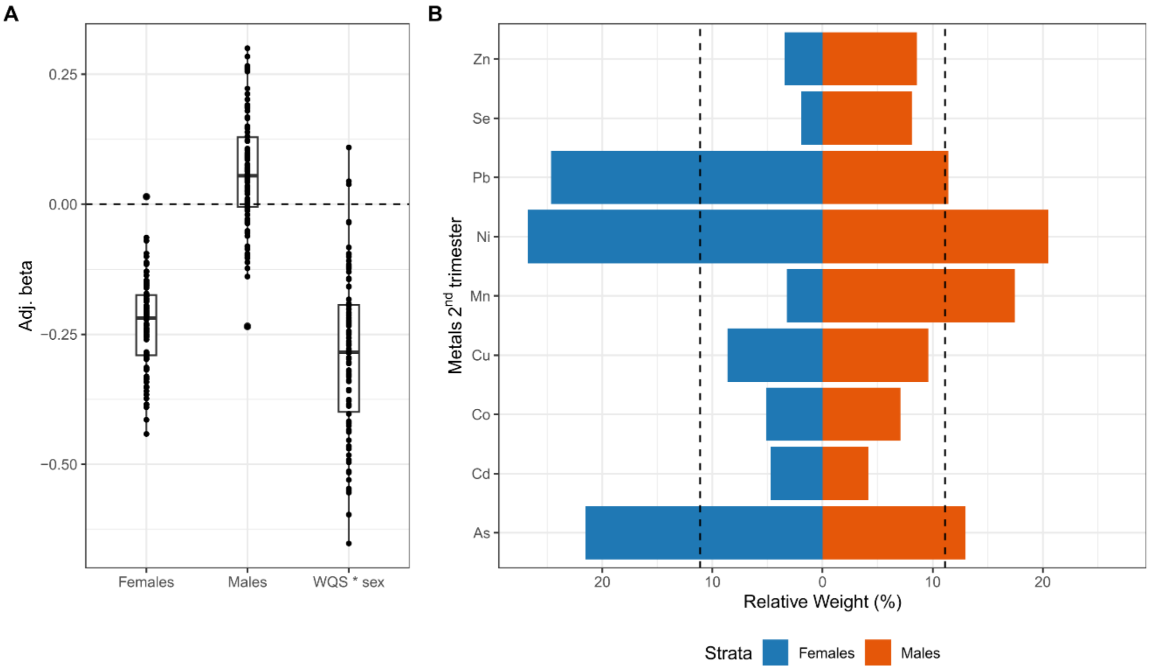

Figure S8. Mean adjusted betas (A) and sex-specific relative weights (B) from a WQS (negative constraint) linear regression with 100 repeated holdouts examining associations between 3rd trimester metals mixtures and FEV_1 z-score. The model was adjusted for maternal age, education, and ETS. Panel A shows beta estimates across holdouts; each dot represents one iteration. Panel B shows average relative weights for each chemical by sex (males/females). Dotted line indicates the threshold (11.11%) for metals of concern. Abbreviations: FEV_1, forced expiratory volume in 1 s; ETS, environmental tobacco smoke

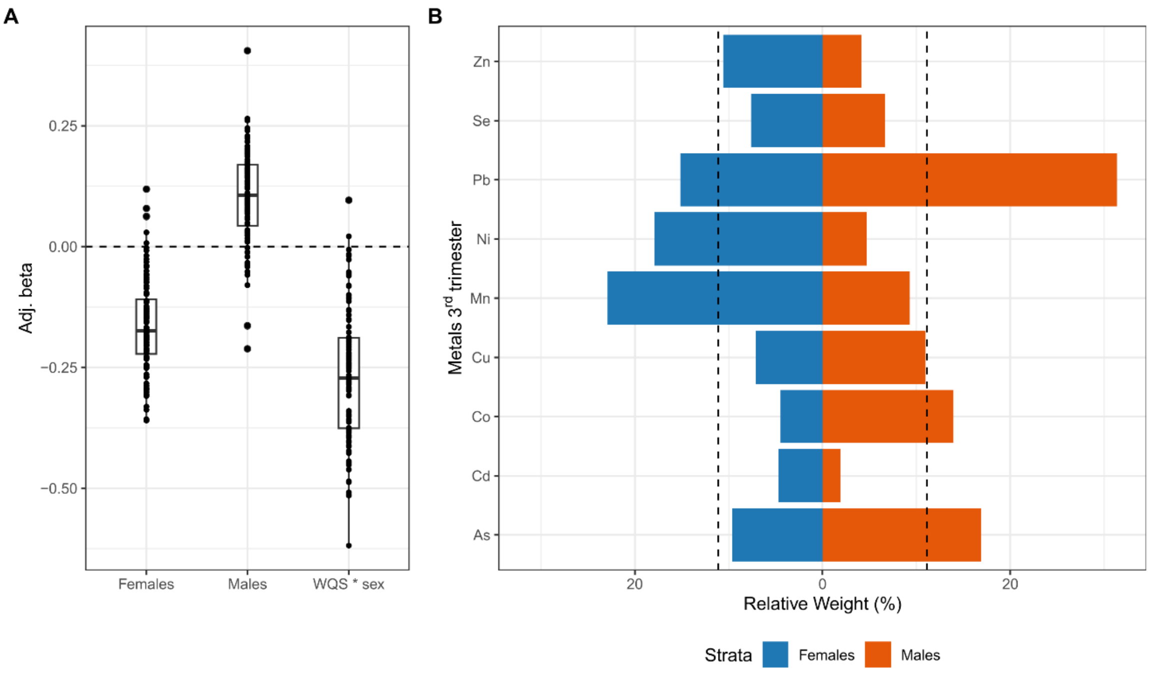

Figure S9. Mean adjusted betas (A) and sex-specific relative weights (B) from a WQS (negative constraint) linear regression with 100 repeated holdouts examining associations between 3rd trimester metals mixtures and FVC z-score. The model was adjusted for maternal age, education, and ETS. Panel A shows beta estimates across holdouts; each dot represents one iteration. Panel B shows average relative weights for each chemical by sex (males/females). Dotted line indicates the threshold (11.11%) for metals of concern. Abbreviations: FVC, forced vital capacity; ETS, environmental tobacco smoke.

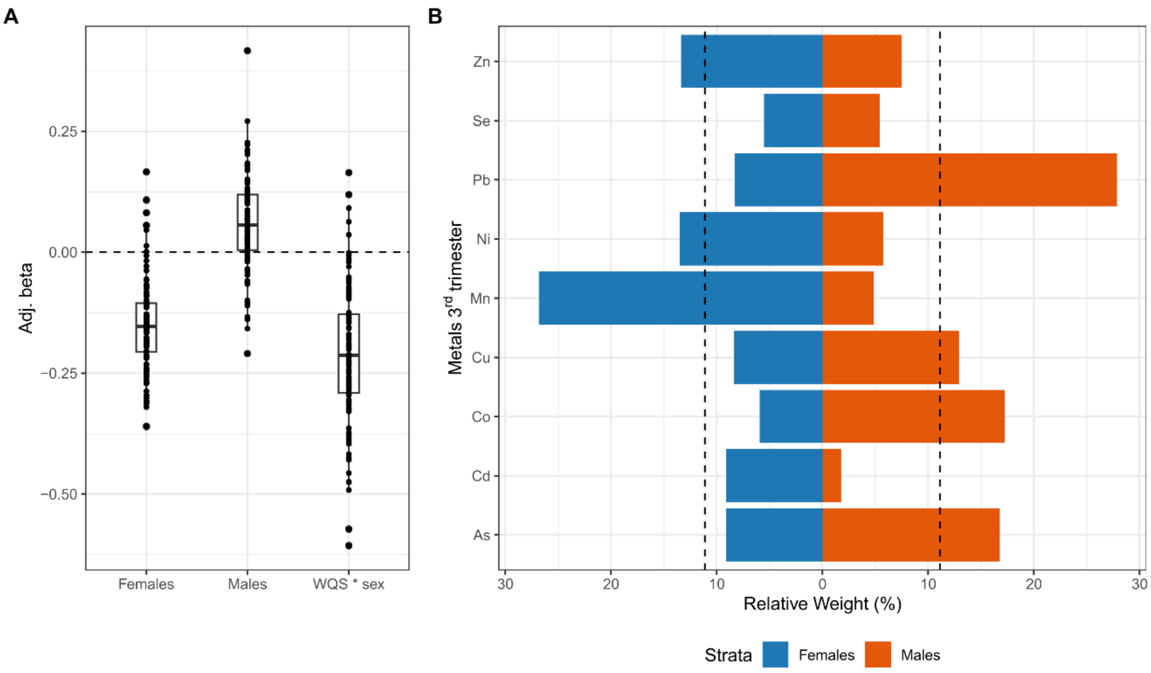

Figure S10. Mean adjusted betas (A) and sex-specific relative weights (B) from a WQS (negative constraint) linear regression with 100 repeated holdouts examining associations between 3rd trimester metals mixtures and FEV_1/FVC ratio. The model was adjusted for maternal age, education, and ETS. Panel A shows beta estimates across holdouts; each dot represents one iteration. Panel B shows average relative weights for each chemical by sex (males/females). Dotted line indicates the threshold (11.11%) for metals of concern. Abbreviations: FEV_1, forced expiratory volume in 1 s; FVC, forced vital capacity; ETS, environmental tobacco smoke.

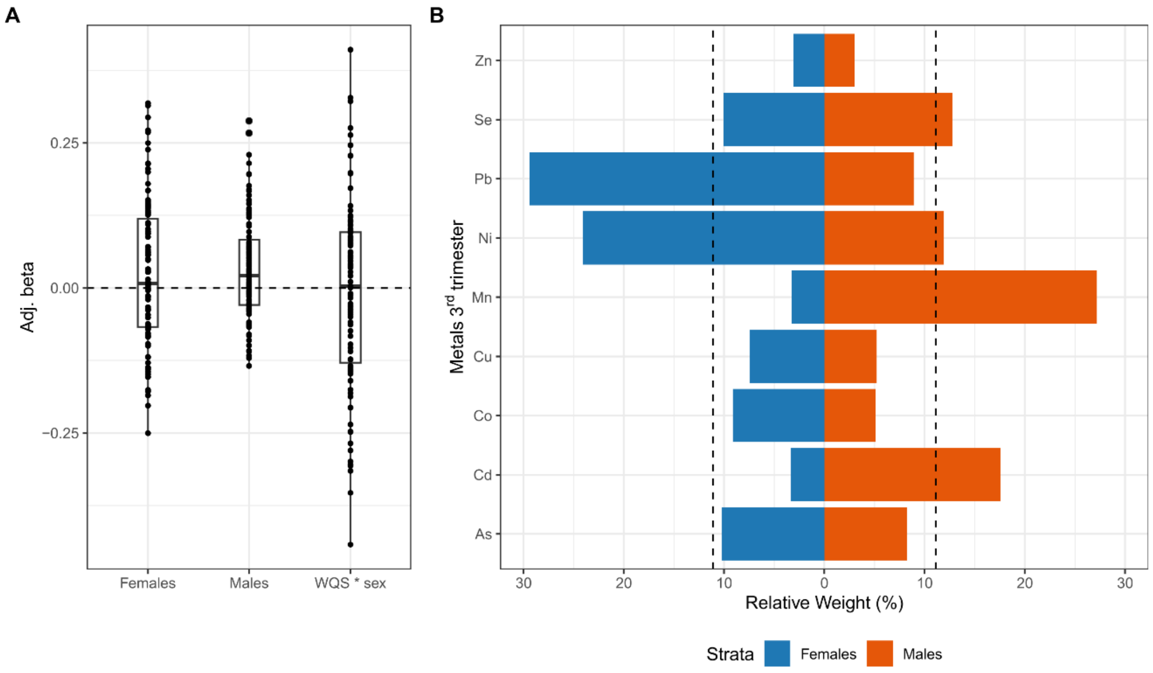

Figure S11. Mean adjusted betas (A) and sex-specific relative weights (B) from a WQS (negative constraint) linear regression with 100 repeated holdouts examining associations between 3rd trimester metals mixtures and $FEF_{25\text{-}75\%}$ z-score. The model was adjusted for maternal age, education, and ETS. Panel A shows beta estimates across holdouts; each dot represents one iteration. Panel B shows average relative weights for each chemical by sex (males/females). Dotted line indicates the threshold (11.11%) for metals of concern. Abbreviations: $FEF_{25–75\%}$, forced expiratory flow between 25% and 75%; FVC, forced vital capacity; ETS, environmental tobacco smoke.

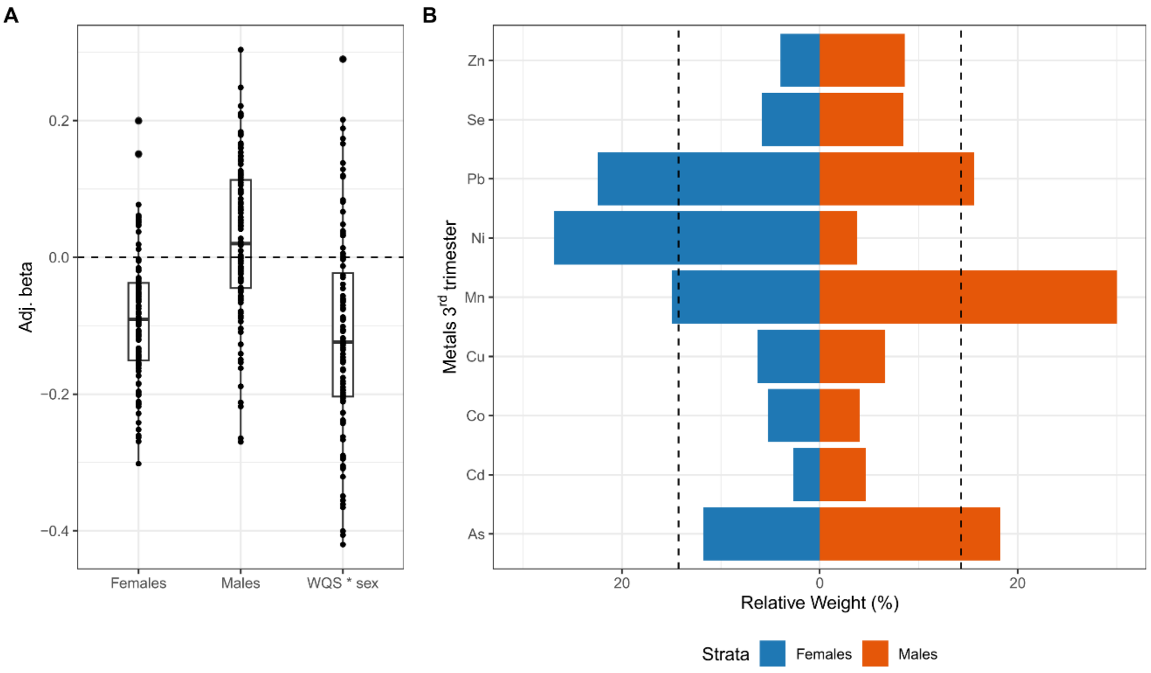

Figure S12. Hierarchical BKMR mixture sex-specific associations between 2nd trimester metals with FEV_1/FCV ratio. The model was adjusted for maternal age, education, ETS, birthweight z-score, gestational age, and preterm birth. Univariate exposure–response plots with 95% credible intervals (A) and cumulative mixture association with FEV_1/FVC ratio (B). Abbreviations: FEV_1, forced expiratory volume in 1 s; FVC, forced vital capacity; ETS, environmental tobacco smoke.

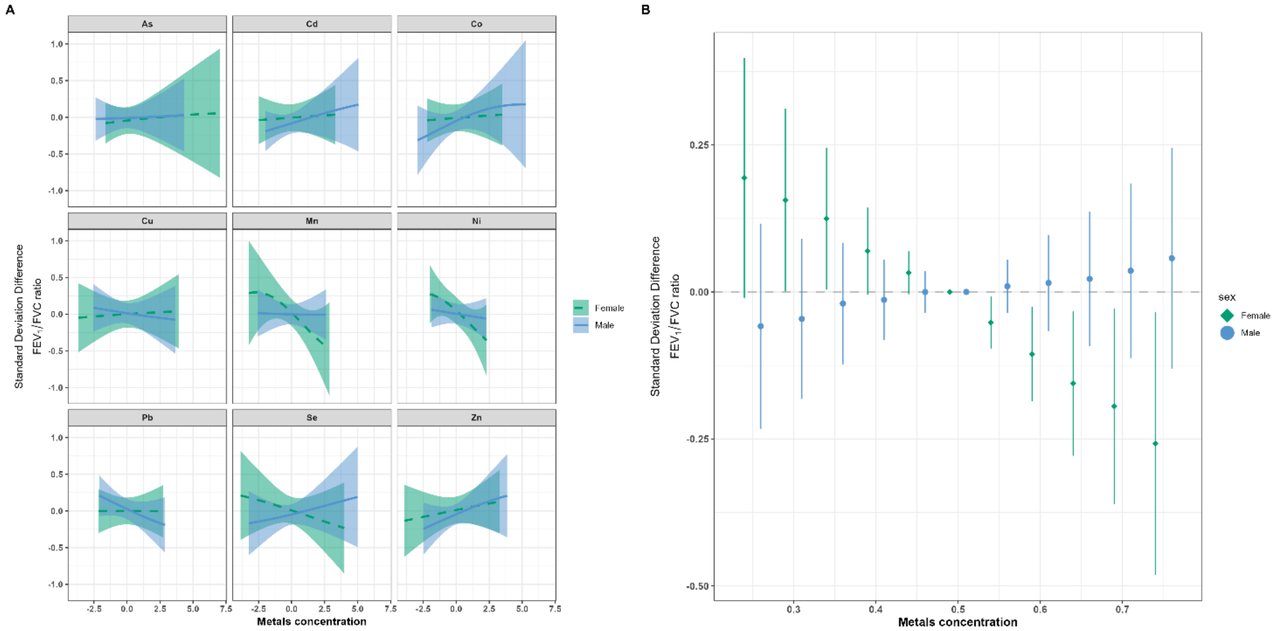

Figure S13. Hierarchical BKMR mixture sex-specific associations between 2nd trimester metals with FEV_1 z-score. The model was adjusted for maternal age, education, and ETS. Univariate exposure–response plots with 95% credible intervals (A) and cumulative mixture association with FEV_1 z-score (B). Abbreviations: FEV_1, forced expiratory volume in 1 s; ETS, environmental tobacco smoke.

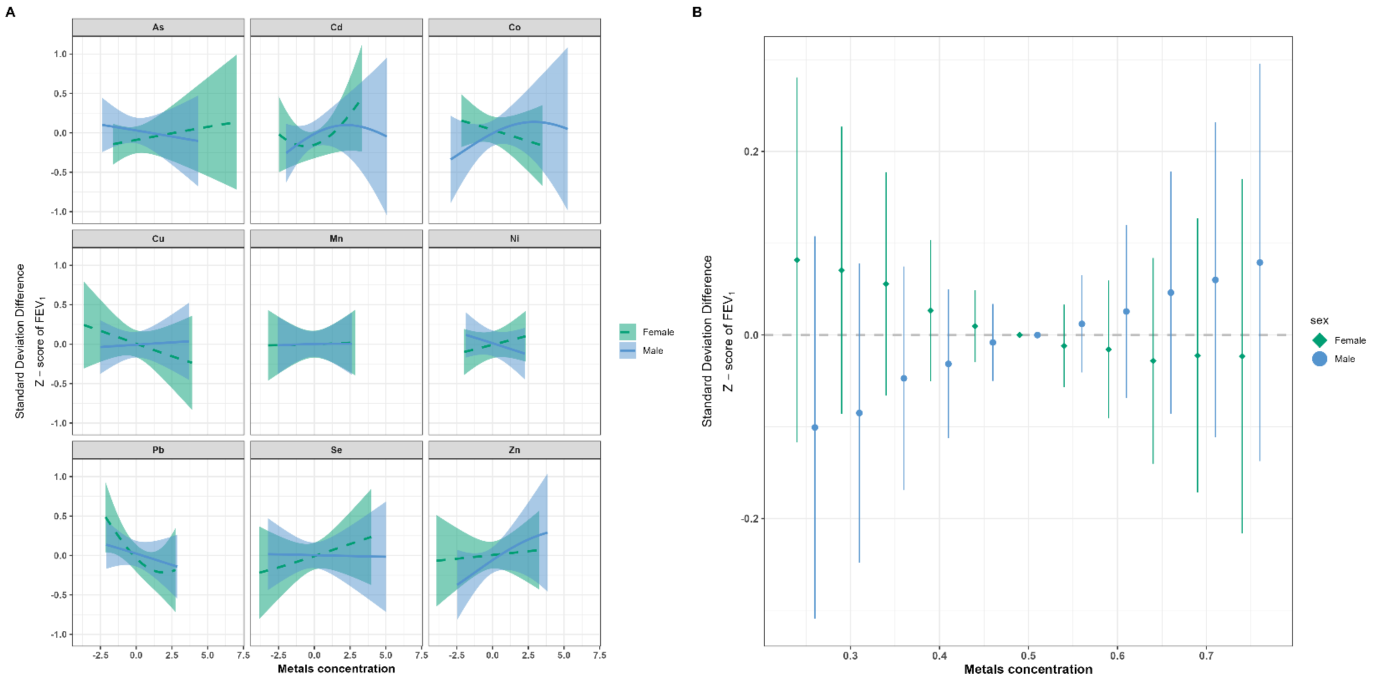

Figure S14. Hierarchical BKMR mixture sex-specific associations between 2nd trimester metals with FVC z-score. The model was adjusted for maternal age, education, and ETS. Univariate exposure–response plots with 95% credible intervals (A) and cumulative mixture association with FVC z-score (B). Abbreviations: FVC, forced vital capacity; ETS, environmental tobacco smoke.

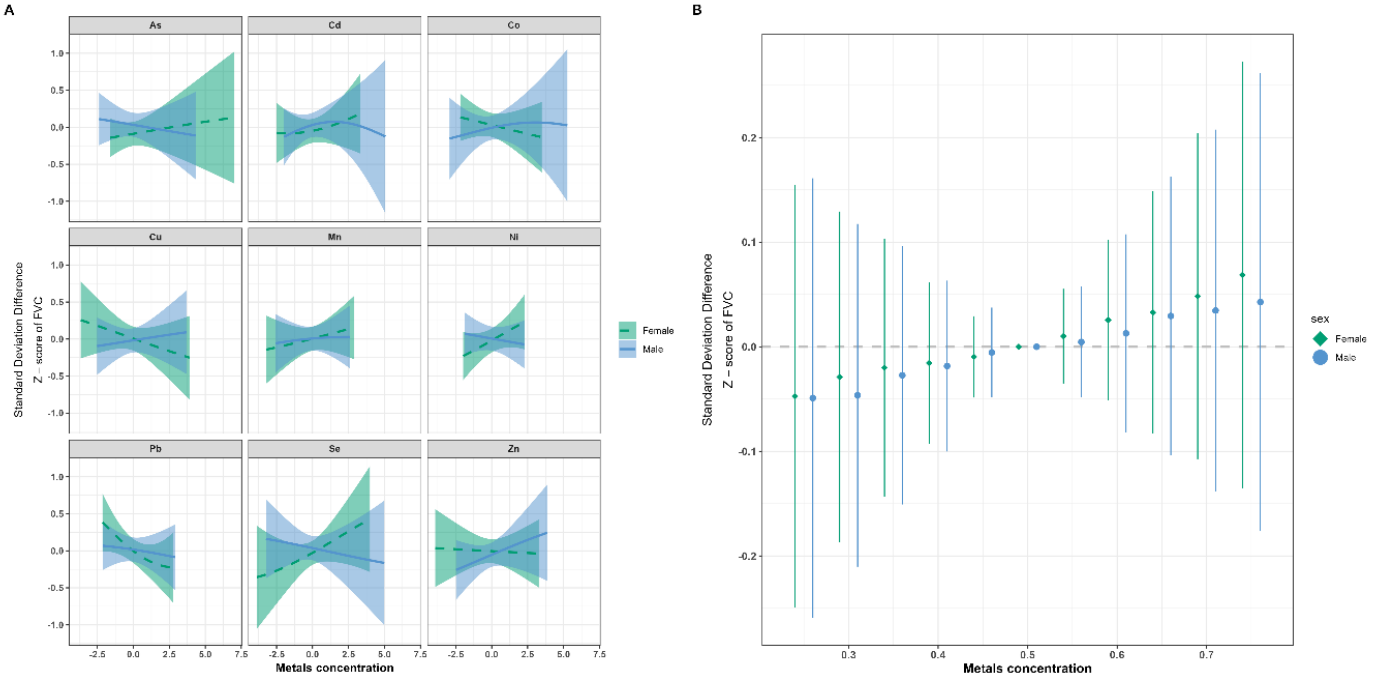

Figure S15. Hierarchical BKMR mixture sex-specific associations between 2nd trimester metals with FEF$_{25\text{-}75\%}$ z-score. The model was adjusted for maternal age, education, and ETS. Univariate exposure–response plots with 95% credible intervals (A) and cumulative mixture association with FEF$_{25\text{-}75\%}$ z-score (B). Abbreviations: FEF$_{25\text{–}75\%}$, forced expiratory flow between 25% and 75%; ETS, environmental tobacco smoke.

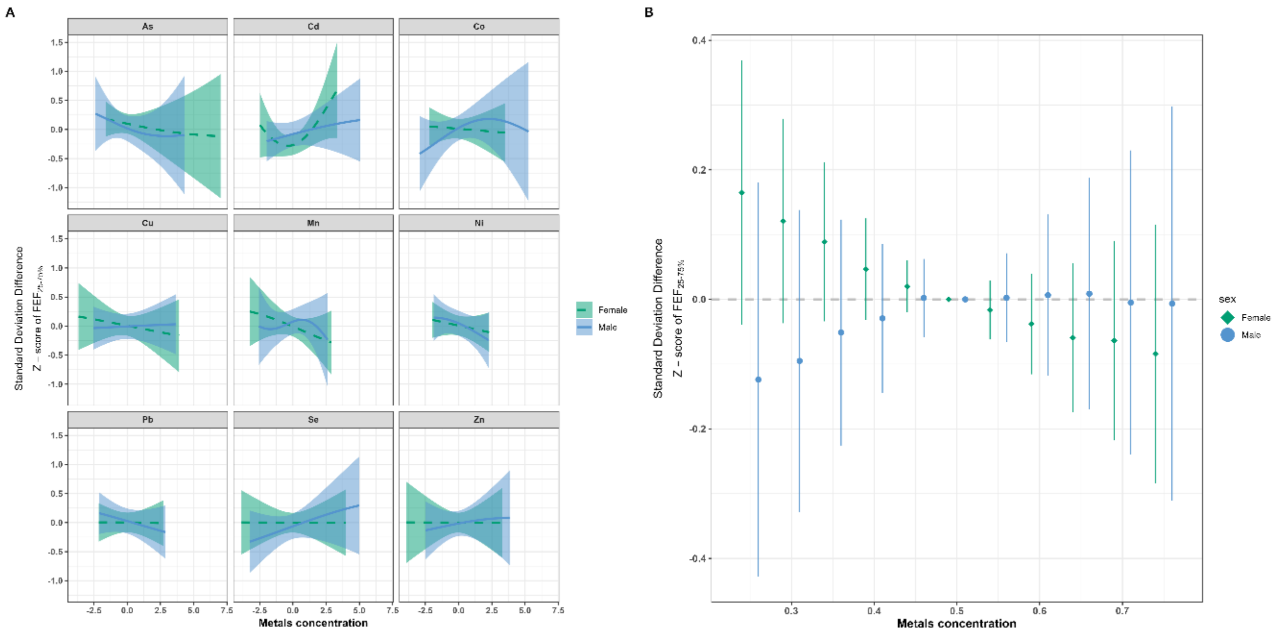

Figure S16. Hierarchical BKMR mixture sex-specific associations between 3rd trimester metals with FEV_1 z-score. The model was adjusted for maternal age, education, and ETS. Univariate exposure–response plots with 95% credible intervals (A) and cumulative mixture association with FEV_1 z-score (B). Abbreviations: FEV_1, forced expiratory volume in 1 s; ETS, environmental tobacco smoke.

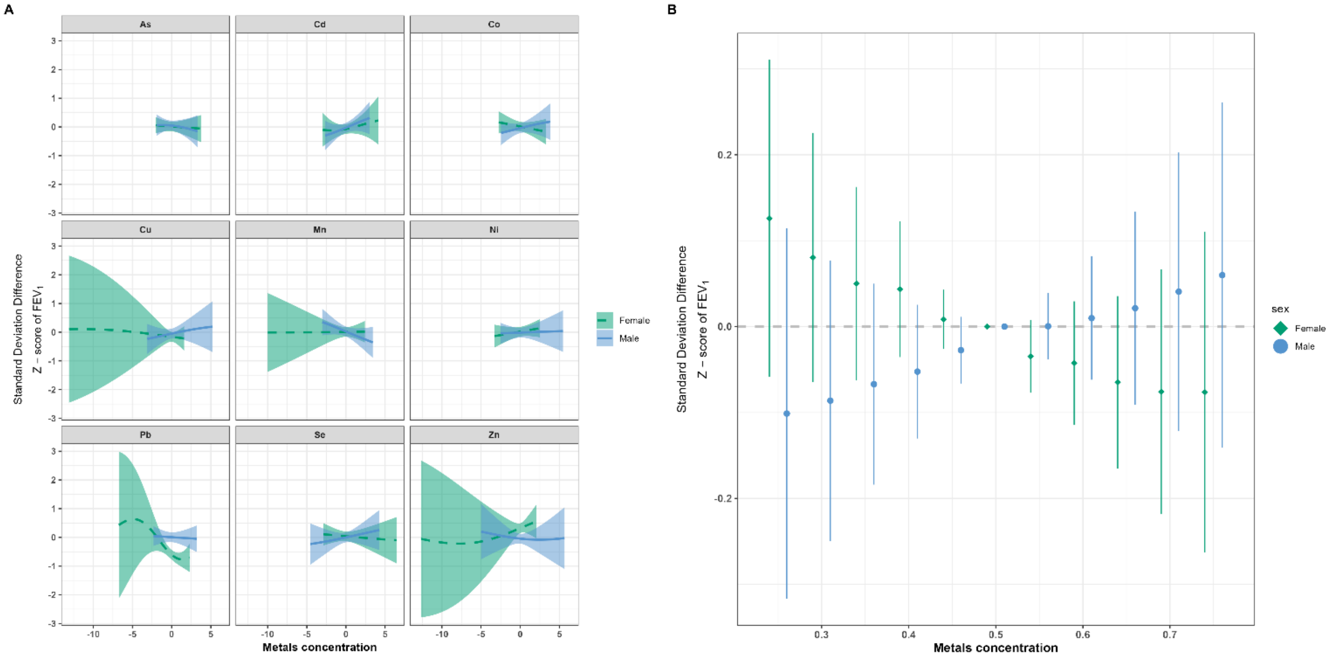

Figure S17. Hierarchical BKMR mixture sex-specific associations between 3rd trimester metals with FVC z-score. The model was adjusted for maternal age, education, and ETS. Univariate exposure–response plots with 95% credible intervals (A) and cumulative mixture association with FVC z-score (B). Abbreviations: FVC, forced vital capacity; ETS, environmental tobacco smoke.

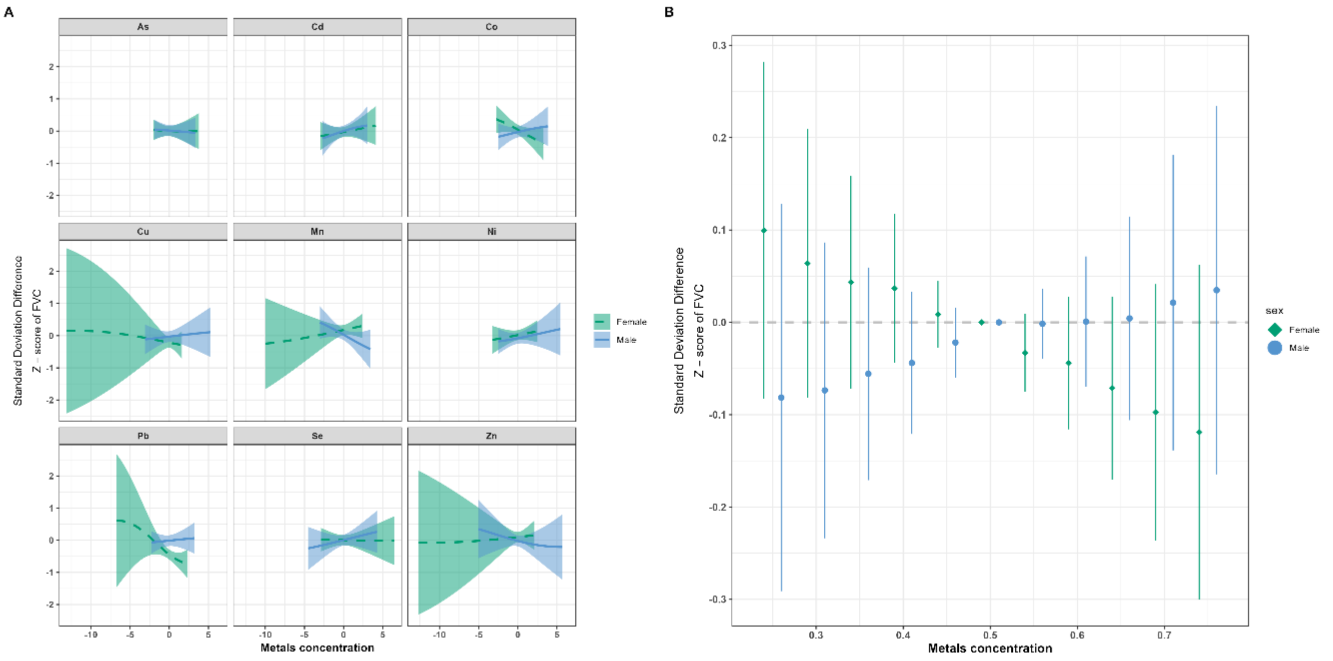

Figure S18. Hierarchical BKMR mixture sex-specific associations between 3rd trimester metals with FEV_1/FVC ratio. The model was adjusted for maternal age, education, and ETS. Univariate exposure–response plots with 95% credible intervals (A) and cumulative mixture association with FEV_1/FVC ratio (B). Abbreviations: FEV_1, forced expiratory volume in 1 s; FVC, forced vital capacity; ETS, environmental tobacco smoke.

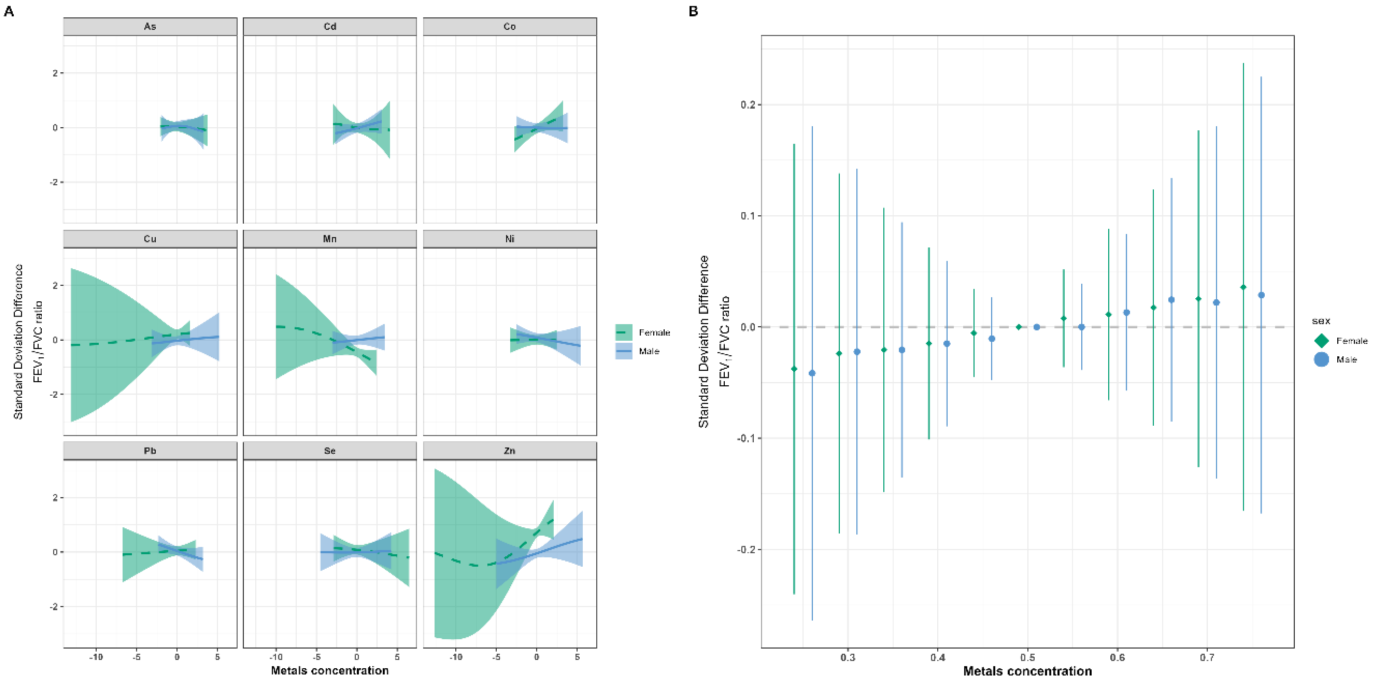

Figure S19. Hierarchical BKMR mixture sex-specific associations between 3rd trimester metals with $FEF_{25-75\%}$ z-score. The model was adjusted for maternal age, education, and ETS. Univariate exposure–response plots with 95% credible intervals (A) and cumulative mixture association with $FEF_{25-75\%}$ z-score (B). Abbreviations: $FEF_{25-75\%}$, forced expiratory flow between 25% and 75%; ETS, environmental tobacco smoke.

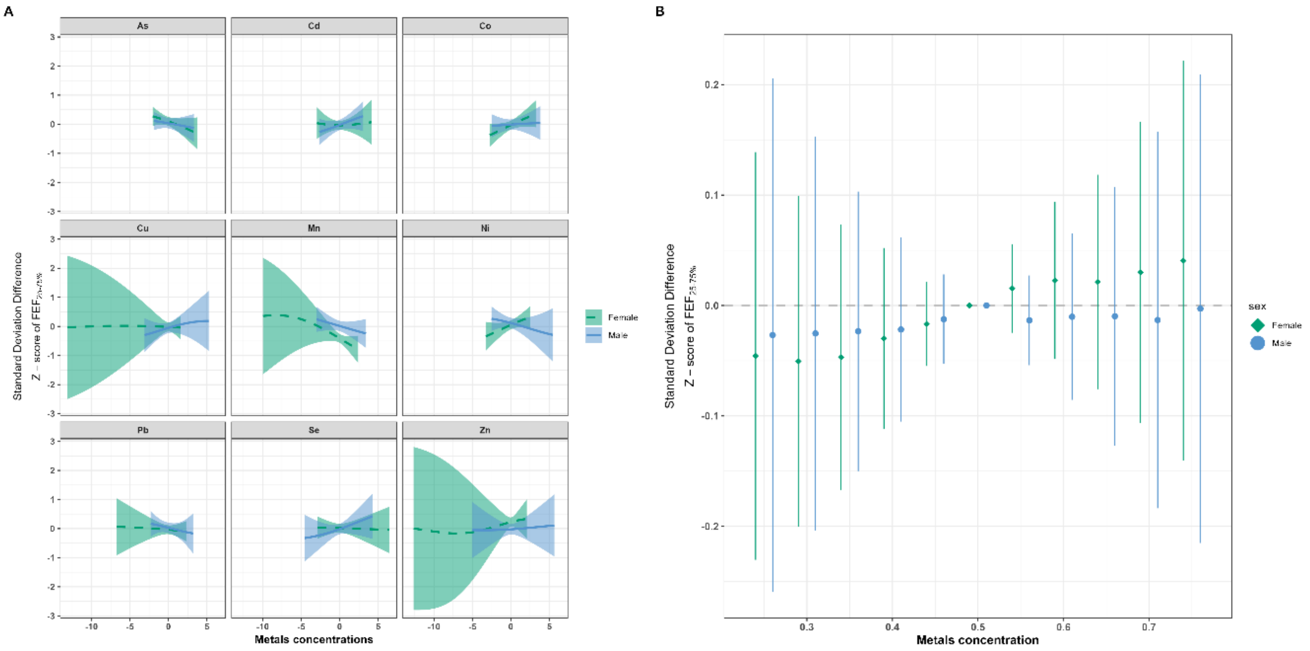

Supplement: Supplementary file 1 [file ee9-9-e447-s001.pdf]
